# Comparison of the conditional approvals for anticancer drugs supported by single-arm trials in China and the United States: clinical evidence, post-marketing requirements, and regulatory outcomes

**DOI:** 10.3389/fphar.2026.1734754

**Published:** 2026-04-10

**Authors:** Yang Du, Shuo Zhang, Li Yang

**Affiliations:** School of Business Administration, Shenyang Pharmaceutical University, Shenyang, China

**Keywords:** antineoplastic drugs, comparative study, drug approval, regulatory science, response rate, single-arm trial

## Abstract

**Purpose:**

Describe and compare the clinical evidence, post-marketing requirements, and regulatory outcomes of all anticancer drugs that have obtained conditional or accelerated approval based on single-arm trial data in China and the United States.

**Methods:**

This paper took all the anticancer drugs that had received accelerated or conditional approval based on single-arm trials in the United States and China as of August 31, 2025, as a research sample, and conducted a comparative analysis of the approval status based on single-arm trials in the United States and China from three aspects: the clinical evidence for the accelerated or conditional approval, the conditions attached to the marketing, and the regulatory outcomes.

**Results:**

Regarding clinical evidence, the phase and number of pivotal clinical trials and sample size in China and the United States were similar, with significant differences in geographic distribution and supporting evidence. In terms of the conditions attached to marketing, there were significant differences between the United States and China in the purpose and confirmatory trial design requirements. In terms of regulatory outcomes, significant differences existed in the time taken to convert to regular approval (RA) and the response rate (RR) of anticancer drugs converted to regular approval in China and the United States. Among these drugs, those simultaneously granted accelerated or conditional approval in both countries based on single-arm trials experienced a significant approval lag in China. Only two conditional approvals were withdrawn in China. At the same time, there was a statistically significant difference between the RR values of anticancer drugs withdrawn in the United States due to unproven clinical benefits and those withdrawn for other reasons.

**Conclusion:**

There are substantial differences between the United States and China across multiple aspects. Compared with the experience of the United States for more than 30 years, China is still in the initial stage, and it is advisable for China to thoroughly learn from the experiences and lessons of the United States to optimize its conditional approval pathway for anticancer drugs supported by single-arm trials.

## 1 Introduction

Oncological diseases, particularly malignant tumors, are irreversible and serious diseases that endanger human life and health. Conditional approval has accelerated the launch of anticancer drugs, allowing promising anticancer drugs to enter the market as early as possible, prolonging patients' lives and improving their quality of life (Huang and Yuan, 2024).

Much clinical trial evidence supporting conditional approval comes from single-arm trials, which provide patients with early access to effective drug treatments. The efficacy of these drugs may take years to be demonstrated in randomized controlled trials (Huang and Yuan, 2024; Wang et al., 2024). For patients with advanced malignant or rare tumors, the absence of effective treatment options, coupled with a short survival time, conditional approval of drugs based on single-arm trial results may be their only hope for obtaining effective treatment (Zhang et al., 2023). The clinical endpoint used to evaluate efficacy in single-arm trials is typically the RR, encompassing Response Evaluation Criteria in Solid Tumors (RECIST), complete response rate, hematological response (e.g., major cytogenetic response and major molecular response), and other criteria (e.g., the International Myeloma Working Group criteria and the Lugano criteria). Since malignancy is a continuously progressive disease that typically does not regress spontaneously (Agrawal et al., 2023), the tumor remission measured by RR may be attributed to drug activity rather than spontaneous regression of the disease, the placebo effect, or other confounding factors.

Overall survival (OS) and validated patient-reported outcomes (PROs) are essential clinical endpoints for patients in oncology clinical trials (Chen et al., 2019; Elbaz et al., 2024). Most trial-level meta-analyses in oncology have found low correlations between surrogate endpoints and OS (Prasad et al., 2015; Ribeiro et al., 2022; Kim and Prasad, 2016; Haslam et al., 2019; Walia et al., 2022; FDA, 2023), and some drugs approved based on response rate have subsequently failed to demonstrate improvements in OS. These approvals increased patient safety costs and toxicity without corresponding clinical benefits. Therefore, as a surrogate endpoint in clinical trials of oncology drugs, RR has advantages and limitations and needs to be rigorously evaluated for risk and benefit when using it.

Since the establishment of the accelerated approval pathway in 1992, the Food and Drug Administration (FDA) in the United States has continuously explored ways to expedite drug launches. On November 17, 1995, Doxil (doxorubicin hydrochloride) received accelerated approval for the treatment of Kaposi sarcoma in acquired immunodeficiency syndrome (AIDS) patients whose disease had progressed during prior combination chemotherapy or who were intolerant to such therapy. It was the first anticancer drug to receive accelerated approval based on the results of a single-arm trial, and its clinical benefits were subsequently confirmed through confirmatory trials, leading to its successful conversion to regular approval. In March 2023, the FDA issued the Clinical Trial Considerations to Support Accelerated Approval of Oncology Therapeutics Guidance for Industry, explicitly highlighting the limitations of single-arm trials and the factors that must be considered when conducting single-arm trials (FDA, 2023). To this day, single-arm trials are still a commonly used pivotal trial design method for accelerated approval in the United States.

Although China started relatively late exploring the conditional approval of anticancer drugs based on single-arm trials, great progress has been made. In December 2020, the National Medical Products Administration (NMPA) of China issued the Guidance for Clinical Communication on Anti-Tumor Drugs Approval by Single-Arm Trial Before New Drug Application and the Guidance for Clinical Communication on Anti-Tumor Drugs Approval by Single-Arm Trial Before Pivotal Study, aiming to assist applicants in more effectively communicating with the Center for Drug Evaluation (CDE) regarding issues related to single-arm trials. Subsequently, the Technical Guideline on the Applicability of Single-Arm Clinical Trials for Use in Support of Oncology Drug Marketing Applications was promulgated in March 2023, aiming to clarify the current scientific understanding of the applicability of single-arm trials for supporting marketing applications of anticancer drugs. China continuously strengthens the guidance for conditional approval for anticancer drugs supported by single-arm trials.

The objective of this study is to characterize and compare the use of single-arm trials in supporting conditional or accelerated approval of all anticancer drugs in China and the United States. Specifically, this study aims to examine the clinical evidence supporting these approvals, the post-marketing requirements imposed, and the regulatory outcomes observed in both countries.

## 2 Data and methods

### 2.1 Data sources

This study identified the list of all anticancer drugs that received accelerated approval based on single-arm trials and their approval time and approval status from January 1, 1992, to August 31, 2025, through the publicly available information on the United States FDA website^1^. The relevant information on single-arm trials, the number and purpose of conditions attached to the marketing, and the content of confirmatory trials were determined through drug labels and letters in the Drugs@FDA database, and information about pivotal clinical trials was obtained through the clinicaltrials.gov database. The anticancer drugs that have received conditional approval based on single-arm trials in China were all retrieved from the official website of the CDE^2^ of the NMPA and the YAOZH database^3^. Relevant information on pivotal clinical trials and the conditions attached to the post-marketing were obtained through drug review reports and the Dingxiangyuan Insight Database^4^. As some drug review reports in China did not differentiate between post-marketing requirements according to the conditional requirements and other post-marketing requirements, this study used other differentiated drugs as examples for statistical analysis.

### 2.2 Sample selection and inclusion criteria

This study analyzed anticancer drugs that received accelerated or conditional approval with single-arm trials as the primary supportive evidence. The dataset was constructed from three analytically distinct groups.

First, all oncology indications granted accelerated approval by the FDA based on single-arm trials were identified (the United States cohort, n = 161). Second, all oncology indications granted conditional approval by the NMPA based on single-arm trials were identified (the China cohort, n = 105). From these two cohorts, a third subset consisting of drugs present in both—i.e., indications that received conditional or accelerated approval in both countries for the same or similar oncologic conditions—was derived (the dual-approval subset, n = 38).

All included approvals met the following criteria: Therapeutic Area: The indication was for oncology, covering all anticancer drugs (including agents for malignant tumors, benign neoplasms, new drugs, generics, and biosimilars). Regulatory Designation: The drug received “Accelerated Approval” in the United States, or its marketing authorization document in China explicitly stated “Conditional Approval.” Pivotal Trial Design (Key Definition): The primary efficacy evidence supporting the marketing application—specifically, the clinical trial explicitly identified as the basis for approval in the drug evaluation reports—must be derived from a single-arm trial.

Exclusion Criteria: Trial Design Mismatch: Drugs approved primarily on the basis of randomized controlled trials (RCTs) were excluded, even if additional single-arm trials were later conducted. Insufficient Information: Drugs for which the pivotal trial design could not be definitively ascertained from public records were excluded.

### 2.3 Statistical analysis

In this study, frequency (percentage) was used to characterize the distribution of categorical data. The Chi-Square test or Fisher’s Exact test was used to compare the categorical data between the two groups. The nonparametric Mann-Whitney U test was used to compare the time taken for conversion to regular approval, the review time of anticancer drugs that received accelerated or conditional approval based on single-arm trials in both China and the United States, and the RR values of single-arm trials of anticancer drugs that had been converted to regular approval. For the descriptive synthesis and comparison of RR values from the pivotal single-arm trials, this study adhered to the 2020 Preferred Reporting Items for Systematic Reviews and Meta-Analyses (PRISMA) guidelines. A subgroup meta-analysis was conducted with the primary objective of systematically describing and comparing the distribution of RRs across different tumor types between the United States and China, rather than estimating a single pooled effect size for all oncology drugs. Given the inherent clinical heterogeneity in tumor biology and expected treatment effects across different indications, significant heterogeneity in RRs was expected. This heterogeneity was assessed using the I^2^ statistic. A random-effects model was applied *a priori* to account for both within-study sampling error and between-study variation in true treatment effects. Pre-defined subgroup analyses by indication were performed to explore this heterogeneity and provide a descriptive overview of the evidence base supporting approvals in each country. Further subgroup analyses, such as by reason for withdrawal in the United States, were conducted to investigate factors associated with regulatory outcomes. The PRISMA checklist is provided in the Supplementary Table S1.

Two-tailed P < 0.05 was used as the significance level for all the above statistical tests. IBM SPSS Statistics version 27, GraphPad Prism version 9.0, Stata 18, and R 4.3.2 were used to complete the above analyses and figures.

## 3 Results

### 3.1 Overall situation analysis

**FIGURE 1:**
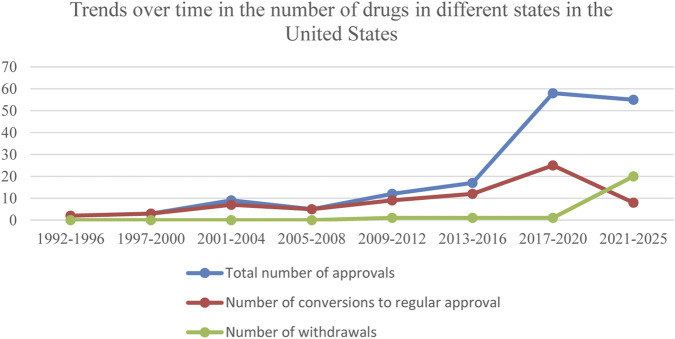
Trends over time in the number of drugs in different states in the United States.

**FIGURE 2:**
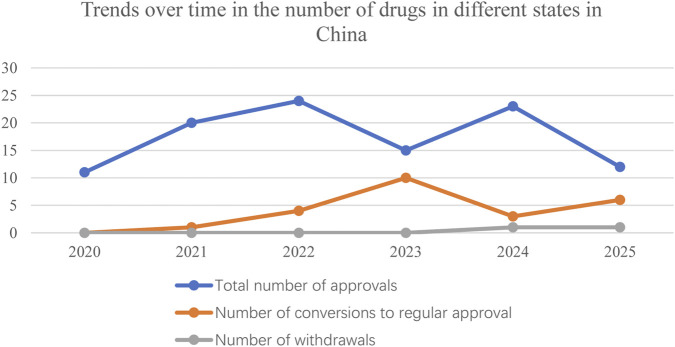
Trends over time in the number of drugs in different states in China.

The line graph showed that the overall trend of anticancer drugs approved in the United States based on single-arm trials was upward, with a relatively flat growth trend from 1992 to 2012 and a significant increase in the growth rate from 2017 to 2020, the number of drugs converted to regular approval also increased, but not as much as the increase in the total number of approvals; and the number of drugs withdrawn from the market increased significantly in the years 2021–2025 (see Figure 1). In China, the number of anticancer drugs approved based on single-arm trials grew annually from 2020 to 2022. Still, there was a significant decline in 2023, with the highest number of anticancer drugs converted to regular approval in 2023 and a slight decrease in 2024. The first drug was withdrawn from the market in China, which did not occur until 2024. As of August 31, 2025, two conditional approvals had been withdrawn (See Figure 2).

As of August 31, 2025, there were 230 accelerated approvals for oncology indications in the United States, of which 161 (70.0%) were successfully approved for marketing based on the results of single-arm trials. In China, there were 121conditional approvals for oncology indications, and 105 (86.8%) of them were approved for marketing based on the results of single-arm trials. The screening and selection process, which involved cross-referencing FDA and NMPA databases with drug labels and review reports, is detailed in the PRISMA flow diagram (Supplementary Figure S1). Of the 161 anticancer drugs in the United States that received accelerated approval based on single-arm trials, 71 (44.1%) had been converted to regular approval, 67 (41.6%) were still under verification, and 23 (14.3%) had been withdrawn; whereas of the 105 anticancer drugs in China that received conditional approval based on single-arm trials, 25 (23.8%) had been successfully converted to regular approval, 78 (74.3%) were still under verification and 2 (1.9%) had been withdrawn. Among the drugs marketed in both the United States and China, both countries had the highest number of drugs under verification, with 22 (57.9%) and 26 (68.4%), respectively. The number of withdrawn drugs was the smallest, with 4 (10.5%) and 1 (2.6%), respectively. See Table 1 for details. In addition, the details of anticancer drugs conditionally approved based on single-arm trials in China and the United States, respectively, are shown in Supplementary Tables S2, S3.

**TABLE 1:** The specific status of obtaining accelerated or conditional approvals based on single-arm trials in the United States and China.

Classifications	Total number of drugs in the US (n = 161) No. (%)	Total number of drugs in China (n = 105) No. (%)	Simultaneously marketed in the United States and in China (n = 38)^a^ No. (%)		P-value^1^	P-value^2^
			United States No. (%)	China No. (%)		
Approval status	​	​	​	​	<0.001	0.369
Converted to RA	71 (44.1)	25 (23.8)	12 (31.6)	11 (29.0)	​	​
Under verification	67 (41.6)	78 (74.3)	22 (57.9)	26 (68.4)	​	​
Withdrawn	23 (14.3)	2 (1.9)	4 (10.5)	1 (2.6)	​	​
Approval indication sequence	​	​	​	​	0.111	0.477
Original	103 (64.0)	77 (73.3)	35 (92.1)	32 (84.2)	​	​
Supplementary	58 (36.0)	28 (26.7)	3 (7.9)	6 (15.8)	​	​
Expedited programs	​	​	​	​	0.025	0.059
BTD	81 (50.3)	30 (28.6)	23 (60.5)	10 (26.3)	​	​
PR	143 (88.8)	93 (88.6)	33 (86.8)	35 (92.1)	​	​

P1: Comparison of approval status distributions between the total United States cohort (n = 161) and the total China cohort (n = 105) using the Chi-Square test or Fisher’s Exact test.

P2: Comparison of approval status distributions between the United States subset (n = 35) and the China subset (n = 35) of drugs approved in both countries, using the Chi-Square test or Fisher’s Exact test.

All P-values were two-sided, and P < 0.05 was considered statistically significant.

Under verification: drugs with ongoing confirmatory trials that have not yet been converted to regular approval.

RA: regular approval.

BTD: breakthrough therapy designation.

PR: priority review.

^a^ The 36 anticancer drugs in China and the 38 in the United States that received conditional or accelerated approvals based on single-arm trials were all the same for the same indication, Among them, Pralsetinib and Selpercatinib were conditionally approved for the treatment of thyroid cancer and medullary thyroid carcinoma in China, and both indications were approved as one approval, whereas the two indications received accelerated approval in the United States, which were two approvals, the rest of the approvals were one-to-one correspondence. For the convenience of the subsequent study, the two indications in China were split, and a one-to-one correspondence with the United States was established to compare and analyze the time and response rate.

### 3.2 The evidence supporting conditional approvals

#### 3.2.1 Status of pivotal clinical trials

Regarding the number of pivotal clinical trials, most approvals in China and the United States were based on one pivotal clinical trial. In addition, in terms of the phase of pivotal clinical trials, most approvals in China and the United States were based on phase II clinical trials. It is important to note that, under standard regulatory pathways, marketing authorization typically requires robust evidence from phase III pivotal trials to confirm both safety and efficacy. However, the drugs analyzed in this study were all approved under the accelerated approval or conditional approval pathways. These pathways are specifically designed to address unmet medical needs in serious or life-threatening diseases, such as advanced malignancies, and allow for approval based on surrogate endpoints from phase II single-arm trials. This regulatory flexibility is intended to expedite patient access to promising therapies while requiring further confirmatory trials post-marketing. Therefore, the reliance on phase II single-arm trials in this context aligns with the intended purpose of these expedited approval programs. In terms of the distribution of clinical trial regions, the United States had a higher proportion of multiregional clinical trials (MRCTs) than China (131 (81.4%) vs. 44 (41.9%), P < 0.001). Regarding the sample size of clinical trials, those with fewer than 100 participants accounted for the largest proportion in both China and the United States. As for the distribution of specific indications, the proportion of approvals for lymphoma in both the United States and China was the highest, with 33 (20.5%) and 27 (25.7%), respectively (Zhang et al., 2022; Luo et al., 2023). In terms of clinical evidence, the most frequent comparisons in both China and the United States were with historical controls, 135 (83.9%) and 70 (66.7%), respectively, while real-world data (RWD) were least applied as external controls. In contrast, China had a higher proportion of target value comparisons than the United States (18 (17.1%) vs. 11 (6.8%), P = 0.005). See Table 2 for details. Additionally, the regulatory requirements for the conditional approval procedures in both China and the United States are provided in Supplementary Table S4.

**TABLE 2:** Status of single-arm Trials and distribution of indications in China and the United States.

Classifications	Total number of drugs in the US (n = 161) No. (%)	Total number of drugs in China (n = 105) No. (%)	P-value
Registration category	​	​	0.696
Chemical drugs	85 (52.8)	58 (55.2)	​
Biologics	76 (47.2)	47 (44.8)	​
No. of pivotal clinical trials	​	​	0.247
1	131 (81.4)	78 (74.3)	​
2	20 (12.4)	21 (20.0)	​
>2	10 (6.2)	6 (5.7)	​
Clinical trial phase	​	​	0.791
I	11 (6.8)	6 (5.7)	​
I/II	34 (21.1)	18 (17.1)	​
II	110 (68.3)	75 (71.4)	​
II/III	2 (1.3)	1 (1.0)	​
III	4 (2.5)	5 (4.8)	​
MRCT	​	​	<0.001
Yes	131 (81.4)	44 (41.9)	​
No	30 (18.6)	61 (58.1)	​
Number of participants in the trials	​	​	0.052
<100	85 (52.8)	71 (67.6)	​
100–200	63 (39.1)	27 (25.7)	​
>200	13 (8.1)	7 (6.7)	​
Classification of indications	​	​	0.232
Lymphoma	33 (20.5)	27 (25.7)	​
Lung cancer	24 (14.9)	23 (21.9)	​
Leukemia	18 (11.2)	9 (8.6)	​
Myeloma	9 (5.6)	8 (7.6)	​
Solid tumors	10 (6.2)	9 (8.6)	​
Urothelial cancer	11 (6.8)	3 (2.8)	​
Colorectal cancer	7 (4.4)	0 (0)	​
Ovarian cancer	5 (3.1)	3 (2.8)	​
Hepatocellular carcinoma	4 (2.5)	1 (1)	​
Other	40 (24.8)	22 (21.0)	​
Supportive evidence	​	​	0.005
Baseline-controlled	13 (8.1)	16 (15.2)	​
RWD-based external control arm	2 (1.2)	1 (1.0)	​
Historical control	135 (83.9)	70 (66.7)	​
Target value	11 (6.8)	18 (17.1)	​

P-value: Comparison of the current status and indication distribution of single-arm trials in China and the United States using the Chi-Square Test or Fisher’s Exact Test.

Baseline-controlled: Baseline-controlled involves comparing the post-intervention data of participants in a single-arm trial with their own pre-intervention values.

Historical control: Comparing with historical data. Historical control data can be derived from the results of a single RCT, a systematic review, or a meta-analysis.

Target value: Comparing with a target value. The target value is determined through a comprehensive analysis of sources such as national standards, industry standards, expert consensus, published literature, research reports, and raw data from relevant studies.

RWD-based external control arm: External controls from real-world data, such as registry data, insurance claims data, and electronic medical records.

#### 3.2.2 Response rates

A meta-subgroup analysis of RR values was performed by categorizing anticancer drugs according to different indications that received accelerated approval based on single-arm trials in the United States and China (Luo et al., 2023; Li et al., 2023; Michaeli et al., 2024). The results showed that the pooled RR values in the United States and China were 40.6% (95% CI: 38.0%–43.5%, I^2^ = 94.7%) and 52.6% (95% CI: 49.2%–56.2%, I^2^ = 93.1%), respectively. There were statistically significant differences in RR values across indications in the United States and China. In the United States, lymphoma had the highest pooled RR value of 57.6% (95% CI: 52.3%–63.6%, I^2^ = 94.1%); hepatocellular carcinoma had the lowest pooled RR value of 22.5% (95% CI: 14.5%–34.9%, I^2^ = 75.7%). In contrast, myeloma in China had the highest pooled RR value of 64.1% (95% CI: 53.3%–77.1%, I^2^ = 95.6%), and uroepithelial carcinoma had the lowest pooled RR value of 32.5% (95% CI: 21.1%–50.1%, I^2^ = 82.3%). The specific results are shown in Supplementary Table S5.

### 3.3 The conditions attached to conditional approval

#### 3.3.1 The number and purpose of conditions

Since 1992, the U.S. FDA had put forward 201 post-marketing requirements for 161 anticancer drugs that received accelerated approval based on single-arm trials, with an average of 1.2 requirements per drug; whereas in China, of the 105 anticancer drugs that received conditional approval based on single-arm trials, 36 had not yet disclosed their review reports, and 97 post-marketing conditions were proposed for the remaining 69 approvals, with an average of 1.4 requirements per drug (Xie et al., 2023; Zhang X. et al., 2024; Mooghali et al., 2024). There were 154 (76.6%) post-marketing requirements in the United States to determine the effectiveness, and 43 (44.3%) in China. However, in the United States, there were only 34 (16.9%) post-marketing requirements for simultaneously determining the efficacy and safety of drugs, while in China, there were 33 (34.0%). From this, it can be seen that compared to the United States, China not only pursues the confirmation of drug effectiveness but also pays more attention to drug safety.

A comparative analysis showed that there were significant differences between the conditions attached to the marketing approval of anticancer drugs based on single-arm trials in China and the United States in terms of confirmation of efficacy, confirmation of safety and efficacy, and supplementation of pharmacokinetic/pharmacodynamic (PK/PD) data. The purpose of other post-marketing conditions attached in the United States was to supplement production process data, while in China, it was to report the progress of clinical studies every year. The details are shown in Table 3.

**TABLE 3:** Number and purpose of conditions attached to marketing approval of anticancer drugs based on single-arm trials in the United States and China.

Conditions attached to marketing approval	The United States (n = 161) No. (%)	China (n = 69) No. (%)	P-value
Confirmation of efficacy	154 (76.6)	43 (44.3)	0.009
Confirmation of safety	3 (1.5)	6 (6.2)	0.063
Confirmation of safety and efficacy	34 (16.9)	33 (34.0)	<0.001
Supplementation of pharmacokinetic/pharmacodynamic (PK/PD) data	9 (4.5)	12 (12.4)	0.013
Other	1 (0.5)	3 (3.1)	0.103
Total	201	97	​

P-value: Comparison of the number and purpose of additional conditions for anticancer drug marketing authorizations based on single-arm trials in the United States and China using the Chi-Square Test or Fisher’s Exact Test.

#### 3.3.2 Confirmatory trials requirements

In the United States, most post-marketing requirements specified the clinical endpoints to be used in confirmatory trials, and only 42 (26.1%) did not require the use of explicit clinical endpoints. Among them, 39 (24.2%) required the use of OS, 45 (27.9%) required the use of progression-free survival (PFS), and 31 (19.3%) required the use of RR. Of the other 4 (2.5%), 2 required Disease-Free Survival (DFS), and 2 required Time to Progression (TTP). At the same time, most of them in China did not specify the clinical endpoints to be used in confirmatory trials, with only 2 explicitly requiring PFS and RR as clinical endpoints.

Regarding the design of confirmatory trials, 109 (67.7%) and 36 (52.2%) of confirmatory trials in the United States and China, respectively, required the use of randomization. Forty-four (27.3%) and 32 (46.4%) required multicenter. Ninety-nine (61.5%) and 32 (46.4%) required control. Fifteen (9.3%) and 11 (15.9%) required the choice of blinding. Twenty-one (13.1%) and 22 (31.9%) explicitly required open-label. Thirty-eight (23.6%) and 4 (5.8%) specified the sample size of the trial. And 32 (19.9%) and 1 (1.4%) requested the duration of follow-up (Xie et al., 2023; Zhang X. et al., 2024; Mooghali et al., 2024). Regarding the time limit for the completion of confirmatory trials (Deshmukh et al., 2023), 58 (36.0%) and 16 (23.2%) of the confirmatory trials in the United States and China, respectively, were required to be completed within 3 years. Thirty-seven (23.0%) and 50 (72.5%) were required to be completed within 3 to 5 years, and 55 (34.2%) and 2 (2.9%) were required to be completed within a time limit of more than 5 years. The details are shown in Table 4.

**TABLE 4:** Requirements for confirmatory trial design after conditional approval of anticancer drugs based on single-arm trials in the United States and China.

Requirements	The United States (n = 161) No. (%)	China (n = 69) No. (%)	P-value
Clinical endpoints	​	​	<0.001
OS	39 (24.2)	0 (0)	​
PFS	45 (27.9)	1 (1.5)	​
RR	31 (19.3)	1 (1.5)	​
Other	4 (2.5)	0 (0)	​
No requirement	42 (26.1)	67 (97.0)	​
Random	​	​	0.025
Yes	109 (67.7)	36 (52.2)	​
No requirement	52 (32.3)	33 (47.8)	​
Multi-center	​	​	​
Yes	44 (27.3)	32 (46.4)	0.005
No requirement	117 (72.7)	37 (53.6)	​
Control group	​	​	0.034
Yes	99 (61.5)	32 (46.4)	​
No requirement	62 (38.5)	37 (53.6)	​
Blinding	​	​	0.007
Yes	15 (9.3)	11 (15.9)	​
No	21 (13.1)	22 (31.9)	​
No requirement	125 (77.6)	36 (52.2)	​
Sample size	​	​	0.001
Yes	38 (23.6)	4 (5.8)	​
No	123 (76.4)	65 (94.2)	​
Follow-up time	​	​	<0.001
Yes	32 (19.9)	1 (1.4)	​
No	129 (80.1)	68 (98.6)	​
Timeframe for submission of research reports	​	​	<0.001
Within 3 years	58 (36.0)	16 (23.2)	​
3–5 years	37 (23.0)	50 (72.5)	​
More than 5 years	55 (34.2)	2 (2.9)	​
No requirement	11 (6.8)	1 (1.4)	​

P-value: Comparison of post-marketing confirmatory trial design requirements for anticancer drugs granted conditional approval based on single-arm trials in the United States and China using the Chi-Square Test or Fisher’s Exact Test.

### 3.4 Regulatory outcomes of conditional approval

#### 3.4.1 Anticancer drugs conditionally approved in both China and the United States

**FIGURE 3:**
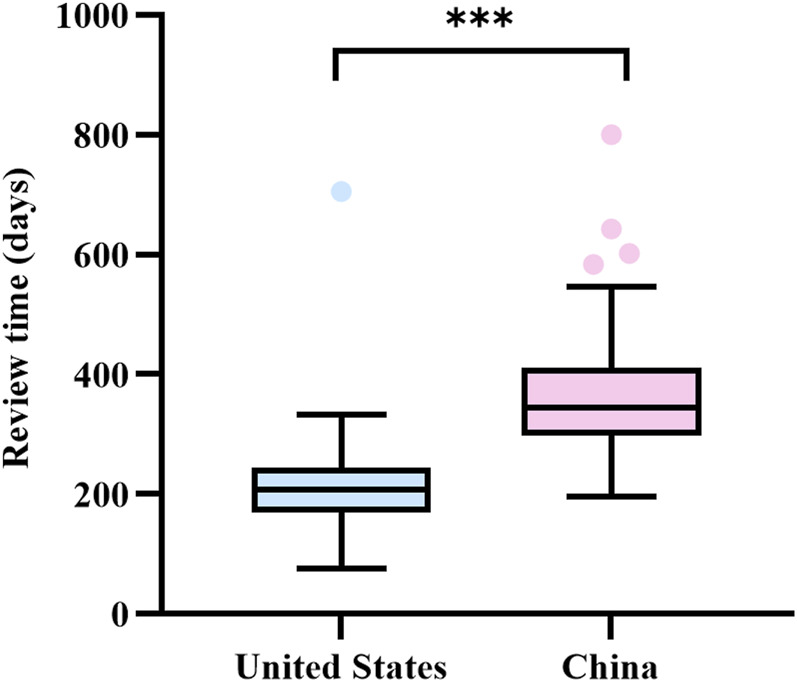
Comparative analysis of review times for simultaneous accelerated or conditional approval based on single-arm trials in China and the United States. Box plots indicate interquartile spacing in shaded areas; short horizontal lines above and below are maximum and minimum values. ^●^ Points are outliers. ***: P < 0.001.

There were 38 anticancer drugs conditionally approved in both China and the United States (see Table 1). A Mann-Whitney U test was performed on the review times of the 38 approvals. The results showed a significant difference in review times for the 38 accelerated approvals in the United States compared to China (see Figure 3). The average review time in the United States was 222 days, with a median time of 208 days (interquartile range: 168.8–243.5). In contrast, the average review time in China was 380 days, with a median time of 344 days (interquartile range: 297.8–410.0). Review times in the United States were shorter than those in China, both in terms of mean and median times. From the point of view of drug approval time, the approval time for drugs in China was later than that in the United States, with an average lag time of 1081 days (3.0 years).

Of these 38 conditional and accelerated approvals, Mobocertinib and Naxitamab were approved for marketing in both China and the United States based on the results of the same international single-arm trials. Furthermore, the response rates of the 36 single-arm trials in China and the United States were slightly different but not statistically significant. Overall, there was no remarkable discrepancy. For the specific drug indications and the response rate values, please refer to Supplementary Table S6.

#### 3.4.2 Anticancer drugs approved in China and the United States

##### 3.4.2.1 Anticancer drugs that have been converted to regular approval

**FIGURE 4:**
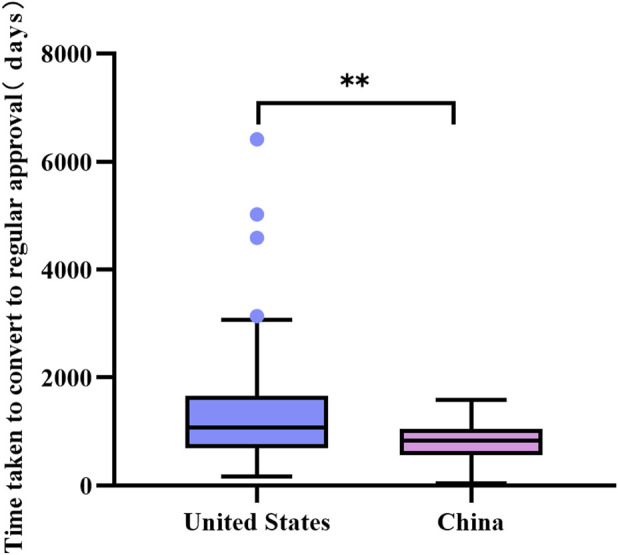
Comparative analysis of the time taken to convert from conditional to regular approval for marketing based on single-arm trials in the United States and China. Box plots indicate interquartile spacing in shaded areas; short horizontal lines above and below are maximum and minimum values. ^●^ Points are outliers. **: P < 0.01.

Seventy-one anticancer drugs in the United States that received accelerated approval based on single-arm trials were successfully converted to regular approval in an average time of 45.4 months (3.8 years), the longest time was 6411 days (17.6 years), and the shortest time was 166 days. While the average conversion time for the 25 approvals in China was 27.4 months (2.3 years), the longest was 1579 days (4.3 years), and the shortest was 42 days. A Mann-Whitney U test was performed on the time taken to convert to regular approval. The results showed a statistically significant difference in the time taken to convert to regular approval after receiving accelerated approval based on a single-arm trial in the United States compared with China (see Figure 4). The median time for the United States to convert to regular approval was longer than that for China (1069 days (interquartile range: 695.0–1659.0) vs. 826 days (interquartile range: 564.0–1049.5), P < 0.01).

**FIGURE 5:**
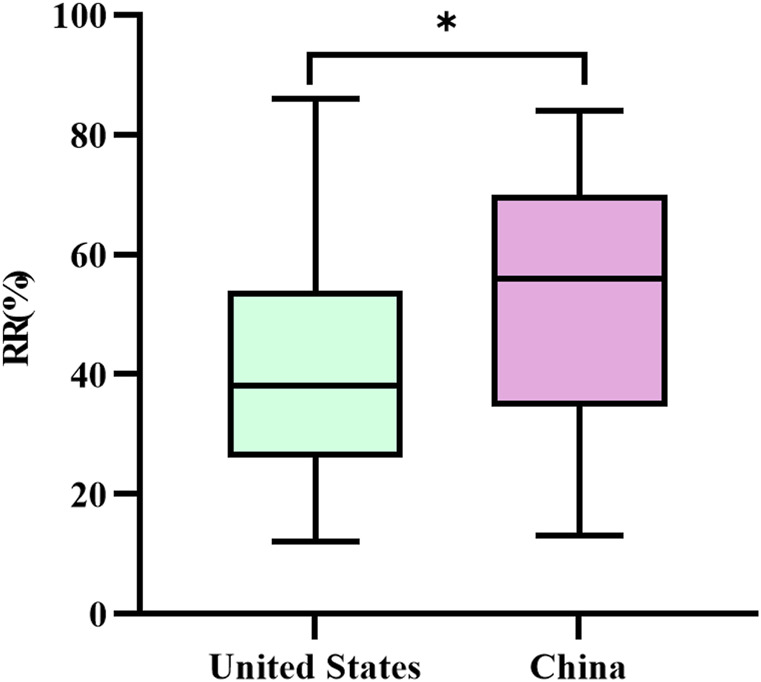
Comparative analysis of response rates in single-arm trials of anticancer drugs that have been converted to regular approval in the United States versus China. Box plots indicate interquartile spacing in shaded areas; short horizontal lines above and below are maximum and minimum values. *: P < 0.05.

For the 69 anticancer drugs in the United States (data for two drugs were unavailable) and 25 anticancer drugs in China, the mean RR values for single-arm trials were 41% and 53%, the maximum RR values were 86% and 84%, and the minimum RR values were 12% and 13%, respectively. There were 22 (31.9%) single-arm trials in the United States, compared with 4 (16.0%) in China, with RR less than 30%, and there were 11 (15.9%) RR values higher than 60% in the United States, compared with 11 (44.0%) in China. A nonparametric Wilcoxon rank-sum test was performed on RR values between the United States and China. The results showed a statistically significant difference in the RR values of the United States single-arm trials compared with China (see Figure 5). The median RR value of the single-arm trials in the United States was lower than that of the single-arm trials in China (38% (interquartile range: 26.0%–54.0%) vs. 56.0% (interquartile range: 34.5%–70.0%), P < 0.05).

According to statistical analysis, there were three forms of conversion pathways between the United States and China for anticancer drugs that have received accelerated or conditional approval based on single-arm trials when converted to regular approval. One was based on the results of a randomized controlled trial, which was the highest in both the United States and China, accounting for 54 (78.3%) and 19 (76.0%), respectively. The second was the continuation of a single-arm trial before accelerated approval, that is, the same trial based on complete trial data, accounting for 11 (16.0%) and 6 (24.0%) in the United States and China, respectively (see Supplementary Table S7). The third was the conversion to regular approval based on the results of a re-conducted single-arm trial (see Supplementary Table S8). This conversion pathway accounted for 4 (5.8%) of the cases in the United States and had not yet appeared in China. The specific endpoints used in the confirmatory trial are shown in Supplementary Table S9. For the requirements for conversion to regular approval in the United States and China, please refer to Supplementary Figure S3.

##### 3.4.2.2 Anticancer drugs under verification

Of the 67 anticancer drugs under verification in the United States, 18 had exceeded the time limit for completion of the confirmatory trial requirements but had not yet been converted to regular approval, accounting for 26.9%; while of the 78 anticancer drugs under verification in China, 4 had exceeded the time limit and had not yet been converted to regular approval, accounting for 5.1%.

##### 3.4.2.3 Anticancer drugs withdrawn

As of August 31, 2025, 31 oncology indications granted accelerated approval in the United States had been withdrawn, of which 23 (74.2%) were based on single-arm trials. The mean time to withdrawal for these 23 anticancer drugs was approximately 1829 days (5.0 years), with the longest and shortest times to withdrawal being 3697 days (10.1 years) and 480 days (1.3 years), respectively. In contrast, only 2 anticancer drugs approved conditionally based on single-arm trials had been withdrawn in China, which was insufficient for comparative analysis with the United States. Accordingly, this study focuses on the analysis of withdrawn anticancer drugs that received accelerated approval based on single-arm trials in the United States.

For the 23 withdrawn anticancer drugs in the United States, the mean RR value of the single-arm trials was 32%, and the maximum and minimum RR values were 66% and 11%, respectively. The median RR value was 24% (interquartile range: 15%–47%). Sixteen (69.6%) accelerated-approval confirmatory trials failed to meet the prespecified clinical endpoints. They thus were withdrawn from the market due to inability to confirm clinical benefits; two (8.7%) were withdrawn because they were unable to enroll enough patients, two (8.7%) were withdrawn because there were safety concerns, and three (13.0%) were withdrawn due to failure to provide the clinical trial data required for post-approval (Koole et al., 2024; Preziosi and Priefer, 2024; Mellgard et al., 2024).

**FIGURE 6:**
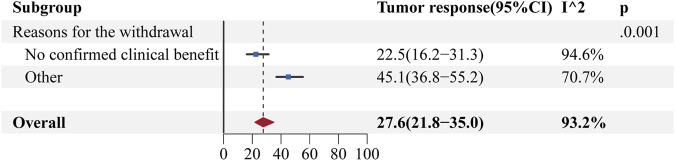
Forest plot of response rates in single-arm trials of withdrawn anticancer drugs in the United States.

According to statistical analysis, most anticancer drugs that received accelerated approval based on single-arm trials were withdrawn because of the inability to demonstrate clinical benefit. A subgroup analysis of RR values was performed to investigate whether there was a significant difference between the RR values of anticancer drugs withdrawn for lack of clinical benefit and those withdrawn for other reasons (Luo et al., 2023; Li et al., 2023; Michaeli et al., 2024). The results showed that all 23 withdrawn approvals had a pooled RR of 27.6% (95% CI: 21.8%–35.0%, I^2^ = 93.2%) for their single-arm trials, whereas the 16 with no proven clinical benefit had a pooled RR of 22.5% (95% CI: 16.2%–31.3%, I^2^ = 94.6%), lower than the remaining 7 with a pooled RR of 45.1% (95% CI: 36.8%–55.2%, I^2^ = 70.7%). This indicated a statistically significant difference between the RR values of anticancer drugs withdrawn because of unproven clinical benefits and those of anticancer drugs withdrawn for other reasons. The results of the specific analysis are shown in Figure 6.

## 4 Discussion

Statistical analysis revealed that, despite the limitations of single-arm trials compared with randomized controlled trials, regulators in both countries have largely granted accelerated or conditional approval for anticancer drugs based on single-arm trial results. However, a recent downward trend has been observed: the number of anticancer drugs receiving accelerated approval based on single-arm trials has decreased in the United States, while approvals in China declined in 2023 and 2025 relative to 2022. This decline is likely driven by two key factors. First, from a regulatory perspective, both countries have implemented stricter control over the conduct of single-arm trials, raising the threshold by issuing technical guidelines: the Clinical Trial Considerations to Support Accelerated Approval of Oncology Therapeutics issued by the FDA and the Technical Guidelines on the Applicability of Single-Arm Clinical Trials for Use in Support of Oncology Drug Marketing Applications issued by the NMPA. Second, from a clinical need perspective, the oncology therapeutic landscape has advanced considerably in recent years. In indications with multiple approved effective therapies, the unmet medical need that previously justified single-arm trials has been partially mitigated. When standard-of-care options are available, conducting a single-arm trial becomes scientifically and ethically more challenging, as randomized controlled trials against active comparators are typically required to demonstrate additional clinical value. While randomized controlled trials are encouraged based on the safety and efficacy of the drug, single-arm trials remain an indispensable pathway to conditional approval where there is an urgent clinical need or randomized controlled trials are not possible. In such cases, more stringent RR requirements should be imposed to mitigate the inherent uncertainty of single-arm trials.

Single-arm trials rely on external controls to interpret treatment effects, with historical controls being the most commonly used in both countries (83.9% in the United States and 66.7% in China) and RWD-based controls the least—an underutilization worthy of regulatory attention amid growing global interest in real-world evidence (RWE). The limited application of RWE stems from practical barriers, including data accessibility challenges, quality concerns, and regulatory acceptance uncertainty, yet RWE offers notable advantages over historical controls: it captures more contemporaneous, representative patient cohorts (in contrast to the restrictive eligibility criteria of historical controls) and can construct better-matched external control arms with advanced methodologies, reducing comparison bias. Recognizing these potential benefits, both NMPA and FDA have issued guidance on RWE. NMPA’s “Guiding Principles for Real-World Evidence to Support Drug Research and Development and Review (Draft)” and the U.S. FDA’s “Considerations for the Use of Real-World Data and Real-World Evidence to Support Regulatory Decision-Making” both encourage the exploration of RWE in regulatory contexts (Alipour-Haris et al., 2024). However, guidance documents alone are insufficient. To realize the potential of RWE as an external control in single-arm trials, collaborative efforts are needed to develop data standards, establish evidentiary thresholds, and build regulatory capacity for evaluating RWE-based submissions. When successfully implemented, RWE-based external controls could address ethical concerns associated with contemporaneous control arms in settings where randomization is challenging, while potentially providing higher-quality evidence than traditional historical comparisons.

Most single-arm trials in both countries enroll fewer than 200 subjects due to practical constraints, yet characterizing a drug’s common adverse reactions and safety profile requires at least 300 patients exposed to the marketing dose—critical data for drug labeling and post-marketing risk management (Department of Commerce of Fujian Province, 2023). Despite this, accelerated or conditional approval pathways prioritize efficacy confirmation in post-marketing requirements, which aligns with their core design of verifying predicted clinical benefits via rigorous post-approval trials. Safety is primarily monitored through post-marketing pharmacovigilance systems and not always formalized in initial approval letters (especially in the United States), while China’s higher proportion of safety-related post-marketing requirements (34.0% vs. 16.9% in the U.S.) reflects a regulatory response to pre-marketing sample size limitations, with a greater emphasis on upfront safety data collection.

Notable differences exist between the United States and China in post-marketing requirements for anticancer drugs granted accelerated or conditional approval based on single-arm trials. In the United States, most post-marketing requirements specify confirmatory trial endpoints and study design, reflecting the greater specificity and complexity of the U.S. regulatory requirements. In contrast, China’s requirements are more streamlined, lacking clarity on confirmatory trial requirements and with no specification of key trial elements such as clinical endpoints—a finding consistent with the early developmental stage of China’s conditional approval pathway (Ge et al., 2024). Anticancer drugs granted conditional approval based on single-arm trials carry considerable uncertainty regarding their true efficacy and full safety profile, underscoring the need for rigorous post-approval confirmatory studies (Cliff et al., 2024). The specific requirements of their post-marketing studies should be clarified to ensure the openness and transparency of anticancer drug studies, which will be more conducive to increasing the research and development impetus of the enterprises and the confidence of the physicians and patients.

Given that most accelerated approvals based on single-arm trials require RCTs to verify clinical benefit, and some trials face considerable implementation challenges, the FDA has permitted longer completion timeframes, with 34.2% of confirmatory trials allocated more than 5 years for completion. In contrast, only 2.9% of confirmatory trials in China were allocated more than 5 years for completion, and most review reports do not specify a definitive timeframe. Notably, the NMPA proposed a principled maximum 4-year completion timeframe for confirmatory trials in its 2023 draft procedure for conditional approval (Department of Comprehensive Affairs, 2023). The NMPA has established a uniform completion timeframe for confirmatory trials across all conditionally approved drugs. However, the feasibility and required duration of confirmatory trials vary substantially across anticancer drugs due to differences in the mechanism of action, therapeutic indication, and study participant characteristics. Therefore, it is suggested that regulatory authorities flexibly specify confirmatory trial completion timeframes based on individual drug characteristics and document these in post-approval requirements to urge enterprises to complete trials on schedule.

For anticancer drugs approved in both countries, China’s approval timing lags significantly behind the United States, driven by longer review times and delayed drug registration dossier submission—the primary barrier to timely approval (Wei et al., 2024). Another key factor is the differential application of expedited regulatory tools: the FDA widely uses Breakthrough Therapy Designation (BTD) and Priority Review (PR) for single-arm trial-based approvals (Table 1), which shorten review cycles via prioritized technical review and on-site inspection. The NMPA has established similar tools (PR, BTD, orphan drug designation, accelerated review), but China’s higher BTD approval threshold and fewer designated anticancer drugs limit the tool’s efficiency in reducing approval time. Despite improvements in China’s drug review efficiency after regulatory reforms, gaps remain in the whole chain of dossier submission, expedited tool application, and review procedure optimization. Thus, the CDE should continuously optimize review procedures to shorten review time and improve evaluation efficiency, while enterprises need to synchronize global clinical research and dossier preparation, and adhere to CDE requirements for registration submissions to avoid unnecessary delays.

Moreover, this study found that the median RR of anticancer drugs granted conditional approval in China based on single-arm trials was higher than that for U.S. drugs. This is primarily because many such anticancer drugs are submitted for marketing in China only after their clinical benefits have been verified in the United States or other regions; some are approved directly based on overseas trial data, while others achieve higher RRs in single-arm trials re-conducted in China owing to pre-confirmed efficacy. Previous studies have shown that the early trial participation rate (4.4%) and synchronous trial participation rate (5.4%) of innovative anticancer drugs in China are relatively low compared to the early trial participation rate (66.1%) and synchronous trial participation rate (89.1%) in the United States (Huang et al., 2022). This indicates that most of the early clinical trials in China were conducted later than those in the United States. This phenomenon may be influenced by both commercial considerations and the scientific necessity of assessing ethnic factors. From a commercial standpoint, global pharmaceutical companies often strategically prioritize the United States for first-in-human trials to facilitate early engagement with the FDA and secure a foothold in a major market. Concurrently, from a regulatory science perspective, the evaluation of ethnic factors is critical. Variations in drug metabolism and tolerance across populations necessitate the inclusion of Chinese patients in clinical development, typically through bridging studies or later-phase MRCTs, to ensure the generalizability of safety and efficacy data to the Chinese population. These strategic and scientific imperatives collectively contribute to the delayed initiation of clinical programs in China. Although this gives China a high level of evidence to ensure the safety and efficacy of the drugs to a certain extent when conditional approval is granted, at the same time, it causes the drugs to have a serious lag in China. In this regard, in addition to the need for Chinese companies to improve the efficiency of clinical research, China’s drug regulators should also encourage drug innovation and improve relevant regulations to attract international companies to invest in innovative drugs in China, creating opportunities for earlier participation in global drug development.

An analysis of withdrawn drugs in the United States found that their median RRs in single-arm trials before accelerated approval were significantly lower than those in single-arm trials of anticancer drugs that were successfully converted to regular approval. This suggests that regulators should raise RR requirements for single-arm trial-based approvals but avoid a one-size-fits-all ban on drugs with low RRs (around 30%). Instead, they should exercise heightened vigilance for such applications and conduct a comprehensive assessment considering the rarity or severity of the indication, trial sample size, response duration, and other specific factors. Even if anticancer drugs with low RRs are conditionally approved following a comprehensive evaluation, proactive safety risk mitigation measures should be implemented, and the expedited drug withdrawal process should be optimized to ensure that drugs that are unable to confirm clinical benefit can be rapidly withdrawn from the market.

A withdrawal process was established in the United States concurrent with the accelerated approval pathway (Zhang S. et al., 2024). However, this process was lengthy and unduly constrained by legal provisions. In December 2022, President Joe Biden signed the Consolidated Appropriations Act, 2023 (U.S. Congress, 2022), which includes the Food and Drug Omnibus Reform Act (FDORA) and establishes a more streamlined withdrawal process to revoke drug approvals when post-approval studies fail to confirm efficacy. Furthermore, the FDA provided a detailed description of the streamlined withdrawal procedures in the “Expedited Program for Serious Conditions—Accelerated Approval of Drugs and Biologics Guidance for Industry” issued on December 5, 2024 (see Supplementary Figure S4 for the specific withdrawal procedures). The new procedure is more transparent and allows applicants to appeal these decisions within an expedited time frame. The NMPA should learn from the experience of the FDA and establish a mature drug withdrawal procedure as early as possible to speed up the withdrawal of substandard drugs.

This study has several limitations. First, the incomplete public release of drug review reports in both countries (as of August 31, 2025) limited data availability and analytical scope—notably, 36 of 105 China’s conditionally approved anticancer drug review reports were unavailable for post-marketing requirement analysis. This introduces potential selection bias, as unpublished reports may differ systematically from the 69 analyzed ones: the findings may under- or overestimate the average number of post-marketing conditions or the stringency of China’s requirements, with the bias direction unascertainable without missing data. Future research should re-evaluate these comparisons as more reports are published. Second, this study did not systematically examine post-marketing safety-related labeling changes, which could serve as a proxy for the adequacy of pre-marketing safety databases. Future studies should investigate whether the limited sample sizes of single-arm trials are correlated with an increased frequency of significant safety updates after approval. Finally, while this study focused on conditional approvals in oncology—one of the most prevalent areas for single-arm trials—other therapeutic domains were not systematically evaluated due to heterogeneity in endpoint definitions. Future research should aim to include a broader range of disease areas to generalize findings.

## 5 Conclusion

This study identifies notable differences between the United States and China in the clinical evidence, post-marketing requirements, and regulatory outcomes associated with conditional/accelerated approval of anticancer drugs supported by single-arm trials. Compared with the over three decades of regulatory experience in the U.S., China remains in the early stages of this process. Therefore, China can draw substantially from the U.S. experiences and lessons to optimize its regulatory pathway for the conditional approval of anticancer drugs based on single-arm trial data.

## Data Availability

The raw data supporting the conclusions of this article will be made available by the authors, without undue reservation.

## Footnotes

1 Food and Drug Administration (FDA). Available online at: https://www.fda.gov.(Accessed September 1, 2025)

2 Center for Drug Evaluation (CDE). Available online at: https://www.cde.org.cn.(Accessed September 1, 2025)

3 YAOZH. YAOZH Database. (2025). Available online at: https://vip.yaozh.com.(Accessed September 1, 2025).

4 Dingxiangyuan Insight Database. (2025). Available online at: http://db.dxy.cn.(Accessed September 1, 2025).
