# Supplementary material for: Comparison of the conditional approvals for anticancer drugs supported by single-arm trials in China and the United States: clinical evidence, post-marketing requirements, and regulatory outcomes
Source: Front Pharmacol. 2026 Apr 10;17:1734754. doi: 10.3389/fphar.2026.1734754 (PMC13105871; doi:10.3389/fphar.2026.1734754)
Supplement: Supplementary file 1 [file DataSheet1.pdf]

Supplementary Material

1 Supplementary Figures and Tables

1.1 Supplementary Figures

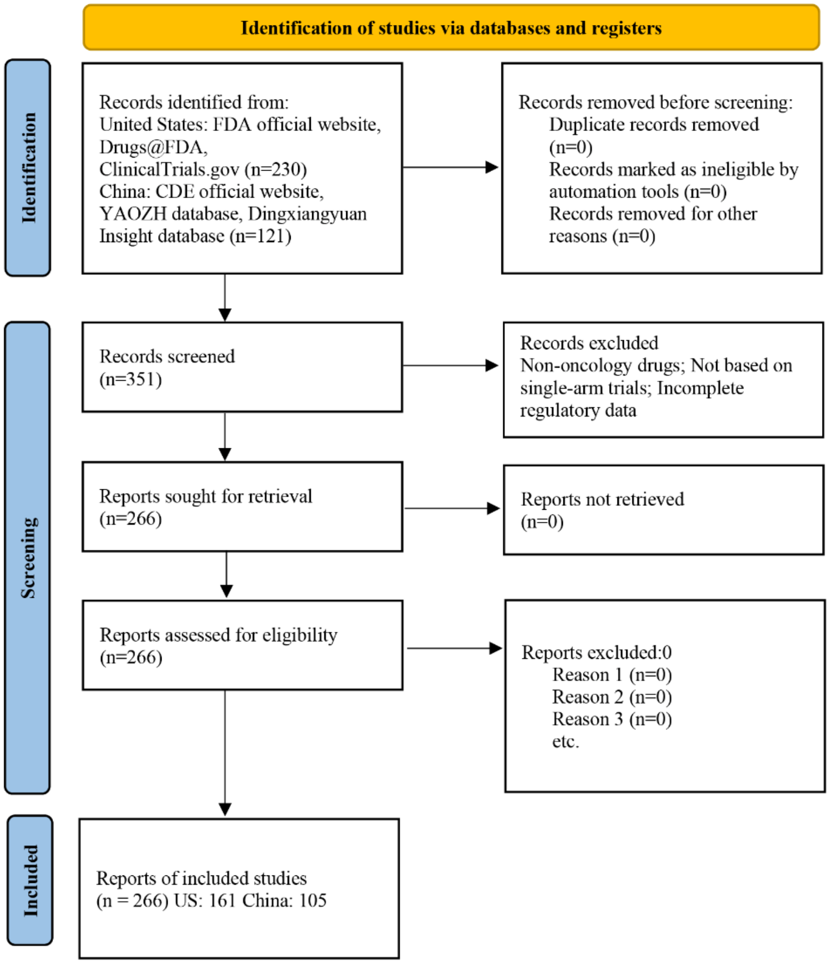

Supplementary Figure 1. PRISMA flow diagram

Product name	Approval Status						Approval time		
	Converted to regular approval		Under verification		Withdrawn		First approval		Approval time difference
	United States	China	United States	China	United States	China	United States	China	
Selinexor	√	√					●		895
Pralatrexate			√	√			●		3989
Mobocertinib					√	√	●		482
Blinatumomab[1]	√			√			●		1490
Blinatumomab[2]	√	√					●		2191
Sacituzumab	√	√					●		776
Carfilzomib	√	√					●		3273
Pemigatinib			√	√			●		711
Naxitamab			√	√			●		735
Duvelisib				√	√		●		1269
Copanlisib				√	√		●		2073
Pralsetinib*			√	√			●		462
Pralsetinib**				√	√		●		462
Pralsetinib***	√	√					●		200
Glofitamab		√	√				●		145
Zanubrutinib		√	√				●		201
Entrectinib			√	√			●		1076
Selpercatinib***	√	√					●		875
Selpercatinib*	√	√					●		875
Selpercatinib**	√	√					●		875
Lorlatinib	√			√			●		1272
Acalabrutinib			√	√			●		1967
Larotrectinib			√	√			●		1229
Selinexor			√	√			●		1467
Teclistamab			√	√			●		602
Pirtobrutinib			√	√			●		634
Pembrolizumab	√	√					●		2296
Mirvetuximab Soravtansine	√			√			●		739
Mosunetuzumab			√	√			●		726
Loncastuximab Tesirine			√	√			●		1323
Lurbinectedin			√	√			●		1630
Zongertinib			√	√			●		18
Elranatamab			√	√			●		568
Talquetamab			√	√			●		549
Zanidatamab			√	√			●		188
Sunvozertinib			√	√				●	-680
Tazemetostat			√	√			●		1734
Tafasitamab			√	√			●		1747

Note:

1 Indications for relapsed or refractory precursor B-cell acute lymphoblastic leukemia in adults and children

2 Indications for adult relapsed or refractory precursor B-cell acute lymphoblastic leukemia

* Indications for thyroid cancer

**Indications for medullary thyroid carcinoma

*** Indications for non-small cell lung cancer

√ Drug approval status in the United States

√ Drug approval status in China

● The drug has been approved for the first time in the United States

● The drug has been approved for the first time in China

Supplementary Figure 2. Approval Status of Conditional Approval for the Same Drug in China and the United States Based on Single-Arm Trials for the Same Indication

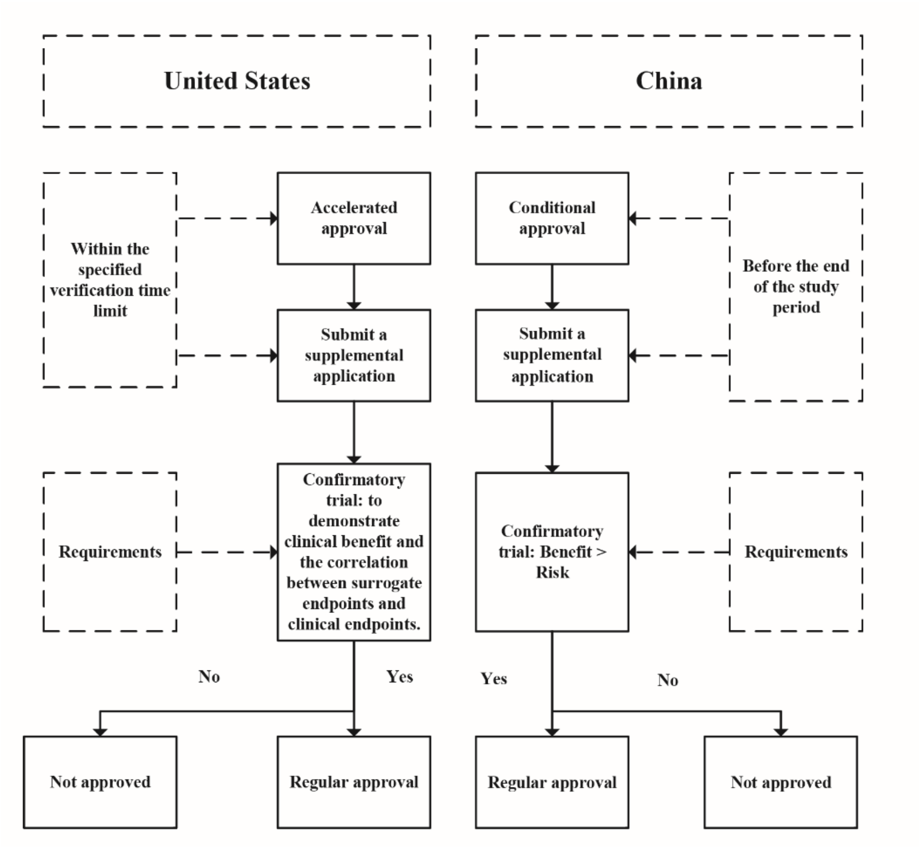

Supplementary Figure 3. Flowchart of the conversion of conditionally approved drugs to regular approval in China and the United States

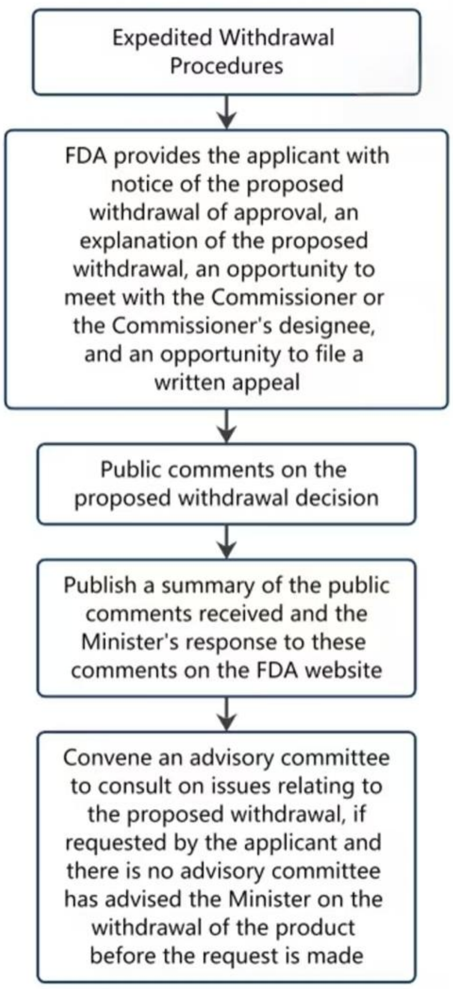

Supplementary Figure 4. Flowchart of the FDA accelerated approval drug withdrawal procedure

1.2 Supplementary Table

Supplementary Table 1. PRISMA checklist

Section and Topic	Item #	Checklist item	Location where item is reported
**TITLE**			
Title	1	Identify the report as a systematic review.	Not Applicable: This article is a comparative policy analysis, not a systematic review. Title: Comparison of the Conditional Approvals for Anticancer Drugs Supported by Single-Arm Trials in China and the United States: Clinical evidence, post-marketing requirements, and regulatory outcomes

**ABSTRACT**			
Abstract	2	See the PRISMA 2020 for Abstracts checklist.	The abstract is structured according to the PRISMA abstract checklist, including the following sections: Objective, Methods, Results, Conclusions.
**INTRODUCTION**			
Rationale	3	Describe the rationale for the review in the context of existing knowledge.	Based on the current landscape of conditional approvals for anticancer drugs supported by single-arm trials in both China and the United States, this article compares the clinical evidence, post-marketing requirements, and regulatory outcomes, with the aim of providing insights for improving the conditional approval pathway in China.
Objectives	4	Provide an explicit statement of the objective(s) or question(s) the review addresses.	This study compares the differences in clinical evidence, post-marketing requirements, and regulatory outcomes for anticancer drugs granted conditional approval based on single-arm trials in China and the United States; it also includes a meta-analysis of response rate (RR) values
**METHODS**			
Eligibility criteria	5	Specify the inclusion and exclusion criteria for the review and how studies were grouped for the syntheses.	Inclusion criteria: All anticancer drugs granted FDA accelerated approval or NMPA conditional approval based on single-arm trials as of August 31, 2025; Exclusion criteria: Non-oncology indications, those not supported by single-arm trials, and drugs without publicly available data.
Information sources	6	Specify all databases, registers, websites, organisations, reference lists and other sources searched or consulted to identify studies. Specify the date when each source was last searched or consulted.	FDA official website, Drugs@FDA, ClinicalTrials.gov, CDE official website, Yaozhi Database, DXY Insight Database
Search strategy	7	Present the full search strategies for all databases, registers and websites, including any filters and limits used.	Not Applicable: This study is a policy analysis, not a systematic literature search.
Selection process	8	Specify the methods used to decide whether a study met the inclusion criteria of the review, including how many reviewers	Screened independently by two authors and cross-checked; any disagreements were adjudicated by a third author.

		screened each record and each report retrieved, whether they worked independently, and if applicable, details of automation tools used in the process.	
Data collection process	9	Specify the methods used to collect data from reports, including how many reviewers collected data from each report, whether they worked independently, any processes for obtaining or confirming data from study investigators, and if applicable, details of automation tools used in the process.	Data were extracted using a standardized form, including drug name, indication, trial phase, sample size, RR values, post-marketing requirements, conversion status, and reasons for withdrawal.
Data items	10a	List and define all outcomes for which data were sought. Specify whether all results that were compatible with each outcome domain in each study were sought (e.g. for all measures, time points, analyses), and if not, the methods used to decide which results to collect.	Primary outcomes: RR values (for meta-analysis), conversion to regular approval, reasons for withdrawal; Secondary outcomes: trial design, post-marketing requirements, review time.
	10b	List and define all other variables for which data were sought (e.g. participant and intervention characteristics, funding sources). Describe any assumptions made about any missing or unclear information.	Drug category, trial phase, sample size, geographic distribution, control type, clinical trial registration information.
Study risk of bias assessment	11	Specify the methods used to assess risk of bias in the included studies, including details of the tool(s) used, how many reviewers assessed each study and whether they worked independently, and if applicable, details of automation tools used in the process.	Not Applicable: This study did not assess the methodological quality of the original studies.

Effect measures	12	Specify for each outcome the effect measure(s) (e.g. risk ratio, mean difference) used in the synthesis or presentation of results.	For RR values only: pooled analysis was performed using a random-effects model; comparisons between groups were conducted using the chi-square test, Fisher's exact test, and Mann-Whitney U test.
Synthesis methods	13a	Describe the processes used to decide which studies were eligible for each synthesis (e.g. tabulating the study intervention characteristics and comparing against the planned groups for each synthesis (item #5)).	For RR values only: a subgroup analysis was performed on all included drugs grouped by indication; a subgroup analysis was performed on withdrawn drugs grouped by reason for withdrawal.
	13b	Describe any methods required to prepare the data for presentation or synthesis, such as handling of missing summary statistics, or data conversions.	For RR values only: a log transformation was performed before pooling, and heterogeneity was assessed using the I^2 statistic.
	13c	Describe any methods used to tabulate or visually display results of individual studies and syntheses.	Box plots and forest plots were generated using GraphPad Prism; meta-analysis was conducted using Stata for RR values only.
	13d	Describe any methods used to synthesize results and provide a rationale for the choice(s). If meta-analysis was performed, describe the model(s), method(s) to identify the presence and extent of statistical heterogeneity, and software package(s) used.	For RR values only: a meta-analysis was performed using a random-effects model, and subgroup analyses were stratified by indication and reason for withdrawal.
	13e	Describe any methods used to explore possible causes of heterogeneity among study results (e.g. subgroup analysis, meta-regression).	For RR values only: subgroup analysis.
	13f	Describe any sensitivity analyses conducted to assess robustness of the synthesized results.	Not Applicable.

Reporting bias assessment	14	Describe any methods used to assess risk of bias due to missing results in a synthesis (arising from reporting biases).	Not Applicable.
Certainty assessment	15	Describe any methods used to assess certainty (or confidence) in the body of evidence for an outcome.	Not Applicable.
**RESULTS**			
Study selection	16a	Describe the results of the search and selection process, from the number of records identified in the search to the number of studies included in the review, ideally using a flow diagram.	A total of 161 approvals in the US and 105 approvals in China based on single-arm trials were included.
	16b	Cite studies that might appear to meet the inclusion criteria, but which were excluded, and explain why they were excluded.	None.
Study characteristics	17	Cite each included study and present its characteristics.	See Tables 1-4 for a detailed list of drug names, indications, trial designs, and RR values.
Risk of bias in studies	18	Present assessments of risk of bias for each included study.	Not Applicable.
Results of individual studies	19	For all outcomes, present, for each study: (a) summary statistics for each group (where appropriate) and (b) an effect estimate and its precision (e.g. confidence/credible interval), ideally using structured tables or plots.	For RR values only: Pooled RR results are presented in Supplementary Table 2; subgroup analyses are shown in Figure 6.
Results of syntheses	20a	For each synthesis, briefly summarise the characteristics and risk of bias among contributing studies.	Not Applicable.

	20b	Present results of all statistical syntheses conducted. If meta-analysis was done, present for each the summary estimate and its precision (e.g. confidence/credible interval) and measures of statistical heterogeneity. If comparing groups, describe the direction of the effect.	For RR values only: Pooled RR for China and the US: 40.6% vs 52.6% (see Supplementary Table 1); Subgroup analysis of RR for withdrawn drugs: 22.5% in the group without verified clinical benefit vs 45.1% in the group with other reasons (see Figure 6).
	20c	Present results of all investigations of possible causes of heterogeneity among study results.	For RR values only: Subgroup analyses indicated that indication and reason for withdrawal were sources of heterogeneity.
	20d	Present results of all sensitivity analyses conducted to assess the robustness of the synthesized results.	Not Applicable.
Reporting biases	21	Present assessments of risk of bias due to missing results (arising from reporting biases) for each synthesis assessed.	Not Applicable.
Certainty of evidence	22	Present assessments of certainty (or confidence) in the body of evidence for each outcome assessed.	Not Applicable.
**DISCUSSION**			
Discussion	23a	Provide a general interpretation of the results in the context of other evidence.	Significant differences exist between China and the US in multiple aspects of conditional approvals based on single-arm trials, with China still in the exploratory stage. Furthermore, a meta-analysis of RR values showed that among withdrawn drugs, the RR value in the group without verified clinical benefit was significantly lower than that in the group with other reasons for withdrawal.
	23b	Discuss any limitations of the evidence included in the review.	Drug review reports for some drugs were not publicly available, which may affect the integrity of the data.
	23c	Discuss any limitations of the review processes used.	Only oncology indications were included; other disease areas were not covered.

	23d	Discuss implications of the results for practice, policy, and future research.	It is recommended that China draw on the US experience to improve the conditional approval pathway, clarify post-marketing requirements, and establish a rapid withdrawal mechanism; enhanced regulatory vigilance should be applied to drugs with lower RR values.
**OTHER INFORMATION**			
Registration and protocol	24a	Provide registration information for the review, including register name and registration number, or state that the review was not registered.	Not registered.
	24b	Indicate where the review protocol can be accessed, or state that a protocol was not prepared.	Not Applicable.
	24c	Describe and explain any amendments to information provided at registration or in the protocol.	Not Applicable.
Support	25	Describe sources of financial or non-financial support for the review, and the role of the funders or sponsors in the review.	This work was supported by the by the Liaoning Province Social Science Planning Fund, China (grant number L25BGL038)
Competing interests	26	Declare any competing interests of review authors.	No conflicts of interest.
Availability of data, code and other materials	27	Report which of the following are publicly available and where they can be found: template data collection forms; data extracted from included studies; data used for all analyses; analytic code; any other materials used in the review.	Data are available from the corresponding author upon reasonable request.

Supplementary Table 2. Status of drugs Accelerated approved only in the United States

Number	Drugs with Accelerated Approval Only in the US	Approval Date	Indication	Approval Status in China
1	Venetoclax	2018/11/21	For the treatment of newly-diagnosed acute myeloid leukemia (AML) in adults who are age 75 years or older, or who have comorbidities that preclude the use of intensive induction chemotherapy, in combination with azacitidine, or decitabine, or low-dose cytarabine	Conditional Approval, based on randomized controlled trial
2	Venetoclax	2016/4/11	For the treatment of patients with chronic lymphocytic leukemia (CLL) with 17p deletion, as detected by an FDA-approved test, who have received at least one prior therapy.	Regular Approval
3	Ibrutinib	2014/2/12	For the treatment of patients with chronic lymphocytic leukemia (CLL) who have received at least one prior therapy.	Regular Approval
4	Ponatinib	2012/12/14	For the treatment of adult patients with chronic phase, accelerated phase, or blast phase chronic myeloid leukemia (CML) that is resistant or intolerant to prior tyrosine kinase inhibitor (TKI) therapy, or Philadelphia chromosome-positive acute lymphoblastic leukemia (Ph+ ALL) that is resistant or intolerant to prior TKI therapy.	Regular Approval
5	Omacetaxine mepesuccinate	2012/10/26	For the treatment of adult patients with chronic or accelerated phase chronic myeloid leukemia (CML) who have resistance and/or intolerance to two or more tyrosine kinase inhibitors (TKIs).	Not Approved
6	Ofatumumab	2009/10/26	For the treatment of patients with chronic lymphocytic leukemia (CLL) refractory to fludarabine and alemtuzumab.	Not Approved
7	Nilotinib	2007/10/29	For the treatment of adult patients with chronic phase and accelerated phase Philadelphia chromosome-positive chronic myeloid leukemia (CML) who are resistant or intolerant to prior therapy, including Gleevec (imatinib).	Regular Approval
8	Imatinib mesylate	2006/9/27	For the treatment of pediatric patients with newly diagnosed Philadelphia chromosome-positive chronic myeloid leukemia (CML).	Not Approved

9	Dasatinib	2006/6/28	For the treatment of adults with chronic myeloid leukemia (CML) with resistance or intolerance to prior therapy including imatinib.	Regular Approval
10	Nelarabine	2005/10/28	For the treatment of patients with T-cell acute lymphoblastic leukemia (T-ALL) and T-cell lymphoblastic lymphoma (T-LBL) whose disease has not responded to or has relapsed following treatment with at least two chemotherapy regimens.	Not Approved
11	Clofarabine	2004/12/28	For the treatment of pediatric patients 1 to 21 years old with relapsed or refractory acute lymphoblastic leukemia (ALL) after at least two prior therapies.	Not Approved
12	Imatinib mesylate	2001/5/10	For the treatment of patients with chronic myeloid leukemia (CML) in blast crisis, accelerated phase, or chronic phase after failure of interferon-alpha therapy.	Not Approved
13	Alemtuzumab	2001/5/7	For the treatment of patients with B-cell chronic lymphocytic leukemia (CLL) who have been treated with alkylating agents and who have failed fludarabine therapy.	Not Approved
14	Imatinib mesylate	2003/5/20	For the treatment of pediatric patients with Philadelphia chromosome-positive chronic phase chronic myeloid leukemia (CML) whose disease has relapsed after stem cell transplant or who are resistant to interferon-alpha therapy.	Not Approved
15	Enfortumab vedotin-efjv	2023/4/3	For the treatment of adult patients with locally advanced or metastatic urothelial cancer (mUC) who are not eligible for cisplatin-containing chemotherapy, in combination with pembrolizumab.	Regular Approval
16	Pembrolizuma b	2023/4/3	For the treatment of adult patients with locally advanced or metastatic urothelial cancer (mUC) who are not eligible for cisplatin-containing chemotherapy, in combination with enfortumab vedotin-ejfv.	Regular Approval
17	Tisotumab vedotin-tftv	2021/9/20	For the treatment of adult patients with recurrent or metastatic cervical cancer with disease progression on or after chemotherapy.	Not Approved
18	Amivantamab -vmjw	2021/5/21	For the treatment of adult patients with locally advanced or metastatic non-small cell lung cancer (NSCLC) with epidermal growth factor receptor (EGFR) exon 20 insertion mutations, as detected by an FDA-approved	Regular Approval

			test, whose disease has progressed on or after platinum-based chemotherapy.	
19	Dostarlimab-gxly	2021/4/22	For the treatment of adult patients with mismatch repair deficient (dMMR) recurrent or advanced endometrial cancer (EC), as determined by an FDA-approved test, that has progressed on or following a prior platinum-containing regimen.	Not Approved
20	Cemiplimab-rwlc	2021/2/9	For the treatment of patients with metastatic basal cell carcinoma (mBCC) previously treated with a hedgehog pathway inhibitor (HHI) or for whom an HHI is not appropriate.	Not Approved
21	Tepotinib	2021/2/3	For the treatment of adult patients with metastatic non-small cell lung cancer (NSCLC) harboring mesenchymal-epithelial transition (MET) exon 14 skipping alterations.	Regular Approval
22	Capmatinib	2020/5/6	For the treatment of adult patients with non-small cell lung cancer (NSCLC) whose tumors have a mutation that leads to MET exon 14 skipping, as detected by an FDA-approved test.	Regular Approval
23	Fam-trastuzumab deruxtecan-nxki	2019/12/20	For the treatment of adult patients with unresectable or metastatic HER2-positive breast cancer who have received two or more prior anti-HER2-based regimens in the metastatic setting.	Regular Approval
24	Enfortumab vedotin-ejfv	2019/12/18	For the treatment of adult patients with locally advanced or metastatic urothelial cancer (mUC) who have received a PD-1 or PD-L1 inhibitor and platinum-containing chemotherapy in the neoadjuvant, adjuvant, or locally advanced or metastatic setting.	Regular Approval
25	Pembrolizumab	2019/9/17	For the treatment of advanced endometrial carcinoma (non-MSI-H or dMMR) in combination with lenvatinib, that has progressed following systemic therapy and is not amenable to curative surgery or radiation.	Not Approved
26	Lenvatinib	2019/9/17	For the treatment of advanced endometrial carcinoma (non-MSI-H or dMMR) in combination with pembrolizumab, that has progressed following systemic therapy and is not amenable to curative surgery or radiation.	Not Approved
27	Erdafitinib	2019/4/12	For the treatment of patients with locally advanced or metastatic urothelial carcinoma (mUC) with susceptible FGFR3 or FGFR2 genetic alterations who have progressed during or following platinum-containing chemotherapy, including within 12 months of	Regular Approval

			neoadjuvant or adjuvant platinum-containing chemotherapy.	
28	Pembrolizumab	2018/12/19	For the treatment of adult and pediatric patients with recurrent locally advanced or metastatic Merkel cell carcinoma (MCC).	Not Approved
29	Pembrolizumab	2018/11/9	For the treatment of patients with hepatocellular carcinoma (HCC) who have been previously treated with sorafenib.	Regular Approval
30	Pembrolizumab	2018/6/13	For the treatment of adult and pediatric patients with refractory primary mediastinal large B-cell lymphoma (PMBCL), or who have relapsed after two or more prior lines of therapy.	Not Approved
31	Pembrolizumab	2018/6/12	For the treatment of patients with recurrent or metastatic cervical cancer with disease progression on or after chemotherapy whose tumors express PD-L1 (CPS ≥ 1) as determined by an FDA-approved test.	Not Approved
32	Pembrolizumab	2017/5/18	For the treatment of patients with locally advanced or metastatic urothelial carcinoma who are not eligible for cisplatin-containing chemotherapy.	Regular Approval
33	Avelumab	2017/5/9	For the treatment of patients with locally advanced or metastatic urothelial carcinoma whose disease progressed during or following platinum-containing chemotherapy, or whose disease progressed within 12 months of neoadjuvant or adjuvant treatment with platinum-containing chemotherapy.	Not Approved
34	Avelumab	2017/3/23	For the treatment of adult and pediatric patients 12 years and older with metastatic Merkel cell carcinoma (MCC).	Not Approved
35	Pembrolizumab	2017/3/14	For the treatment of adult and pediatric patients with refractory classical Hodgkin lymphoma (cHL), or who have relapsed after 3 or more prior therapies.	Not Approved
36	Nivolumab	2017/2/2	For the treatment of patients with locally advanced or metastatic urothelial carcinoma:<br>• whose disease progressed during or following platinum-containing chemotherapy<br>• whose disease progressed within 12 months of neoadjuvant or adjuvant treatment with platinum-containing chemotherapy.	Regular Approval
37	Rucaparib	2016/12/19	For the treatment of advanced ovarian cancer (3) associated with BRCA mutations	Not Approved

			(germline and/or somatic) treated with two or more chemotherapies.	
38	Pembrolizumab	2016/8/5	For the treatment of patients with recurrent or metastatic head and neck squamous cell carcinoma (HNSCC) with disease progression on or after platinum-containing chemotherapy.	Conditional Approval, based on randomized controlled trial
39	Alectinib	2015/12/11	For the treatment of patients with anaplastic lymphoma kinase (ALK)-positive metastatic non-small cell lung cancer (NSCLC) whose disease has progressed on or who are intolerant to crizotinib.	Regular Approval
40	Daratumumab	2015/11/16	For the treatment of patients with multiple myeloma who have received at least three prior lines of therapy (including a proteasome inhibitor and an immunomodulatory agent, or who are double-refractory to a proteasome inhibitor and an immunomodulatory agent).	Regular Approval
41	Osimertinib	2015/11/13	For the treatment of patients with metastatic epidermal growth factor receptor (EGFR) T790M mutation-positive non-small cell lung cancer (NSCLC), as detected by an FDA-approved test, whose disease has progressed on or after EGFR tyrosine kinase inhibitor (TKI) therapy.	Regular Approval
42	Pembrolizumab	2015/10/2	For the treatment of patients with metastatic PD-L1-positive non-small cell lung cancer (NSCLC), as determined by an FDA-approved test, whose disease has progressed on or after platinum-containing chemotherapy.	Regular Approval
43	Nivolumab	2014/12/22	For the treatment of patients with unresectable or metastatic melanoma and disease progression following ipilimumab and, if BRAF V600 mutation positive, a BRAF inhibitor.	Not Approved
44	Olaparib	2014/12/19	For the treatment of patients with advanced ovarian cancer (3) associated with deleterious or suspected deleterious germline BRCA mutations, treated with three or more prior lines of chemotherapy.	Regular Approval
45	Ceritinib	2014/4/29	For the treatment of patients with anaplastic lymphoma kinase (ALK)-positive metastatic non-small cell lung cancer (NSCLC) whose disease has progressed on or who are intolerant to crizotinib.	Regular Approval

46	Crizotinib	2011/8/26	For the treatment of patients with locally advanced or metastatic non-small cell lung cancer (NSCLC) that is anaplastic lymphoma kinase (ALK)-positive as detected by an FDA-approved test.	Regular Approval
47	Brentuximab vedotin	2011/8/19	For the treatment of patients with Hodgkin lymphoma (HL) after failure of autologous stem cell transplant (ASCT) or after failure of at least two prior multi-agent chemotherapy regimens in patients who are not ASCT candidates.	Regular Approval
48	Brentuximab vedotin	2011/8/19	For the treatment of patients with systemic anaplastic large cell lymphoma (sALCL) after failure of at least one prior multi-agent chemotherapy regimen.	Regular Approval
49	Everolimus	2010/10/29	For the treatment of patients with subependymal giant cell astrocytoma (SEGA) associated with tuberous sclerosis complex (TSC) who require therapeutic intervention but are not candidates for curative surgical resection.	Not Approved
50	Bevacizumab	2009/5/5	For the treatment of patients with progressive glioblastoma following prior therapy.	Regular Approval
51	Sunitinib maleate	2006/1/26	For the treatment of advanced renal cell carcinoma (RCC).	Regular Approval
52	Cetuximab	2004/2/12	For the treatment of patients with epidermal growth factor receptor (EGFR)-expressing metastatic colorectal cancer (mCRC) who are refractory to irinotecan-based chemotherapy, in combination with irinotecan.	Regular Approval
53	Bortezomib	2003/5/13	For the treatment of patients with multiple myeloma (MM) who have received at least two prior therapies and have demonstrated disease progression on the last therapy.	Regular Approval
54	Imatinib mesylate	2002/2/1	For the treatment of patients with KIT (CD117)-positive unresectable and/or metastatic malignant gastrointestinal stromal tumors (GIST).	Not Approved
55	Temozolomid e	1999/8/11	For the treatment of adult patients with refractory anaplastic astrocytoma (AA).	Regular Approval
56	Doxorubicin hydrochloride	1999/6/28	For the treatment of patients with metastatic ovarian cancer refractory to paclitaxel- and platinum-based chemotherapy regimens.	Not Approved

57	Capecitabine	1998/4/30	For the treatment of patients with metastatic breast cancer resistant to both paclitaxel and an anthracycline-containing chemotherapy regimen, or resistant to paclitaxel and for whom further anthracycline therapy is contraindicated.	Not Approved
58	Irinotecan hcl trihydrote	1996/6/14	For the treatment of patients with metastatic carcinoma of the colon or rectum whose disease has recurred or progressed following 5-FU-based therapy.	Not Approved
59	Doxorubicin hydrochloride	1995/11/17	For the treatment of patients with AIDS-related Kaposi's sarcoma whose disease has progressed on prior combination chemotherapy or who are intolerant to such therapy.	Regular Approval
60	Dordaviprone	2025/8/6	For the treatment of adult and pediatric patients 1 year of age and older with diffuse midline glioma (DMG) harboring a H3 K27M mutation, whose disease has progressed following prior therapy.	Not Approved
61	Linvoseltamab-gcpt	2025/7/2	For the treatment of adult patients with relapsed or refractory multiple myeloma (RRMM) who have received at least four prior lines of therapy, including a proteasome inhibitor, an immunomodulatory agent, and an anti-CD38 monoclonal antibody.	Not Approved
62	Datopotamab deruxtecan-dlnk	2025/6/23	For the treatment of adult patients with locally advanced or metastatic non-squamous, non-small cell lung cancer (NSCLC) with high c-Met protein overexpression [≥50% of tumor cells with strong (3+) staining], as determined by an FDA-approved test, who have received prior systemic therapy.	Not Approved
63	Telisotuzumab vedotin-tllv	2025/5/14	For the treatment of adult patients with locally advanced or metastatic non-squamous, non-small cell lung cancer (NSCLC) with high c-Met protein overexpression [≥50% of tumor cells with strong (3+) staining], as determined by an FDA-approved test, who have received prior systemic therapy.	Not Approved
64	Avmapki Fakzynja Co-Pack (avutometinib capsules; defactinib tablets)	2025/5/8	For the treatment of adult patients with recurrent low-grade serous ovarian cancer (LGSOC) of KRAS mutation status who have received prior systemic therapy.	Not Approved
65	Zenocutuzumab-zbco	2024/12/4	For the treatment of adult patients with advanced, unresectable, or metastatic non-small cell lung cancer (NSCLC) harboring a	Not Approved

			neuregulin 1 (NRG1) gene fusion, with disease progression on or after prior systemic therapy.	
66	Zenocutuzumab-zbco	2024/12/4	For the treatment of adult patients with advanced, unresectable, or metastatic pancreatic adenocarcinoma harboring an NRG1 gene fusion, with disease progression on or after prior systemic therapy.	Not Approved
67	Afamitresgene autoleucel	2024/8/2	For the treatment of adults with unresectable or metastatic synovial sarcoma who have received prior chemotherapy, are HLA-A*02:01P, -A*02:02P, -A*02:03P, or -A*02:06P positive, and whose tumor expresses the MAGE-A4 antigen as determined by FDA-approved or -cleared companion diagnostic devices.	Not Approved
68	Epcoritamab-bysp	2024/6/26	For the treatment of adult patients with relapsed or refractory follicular lymphoma (FL) after two or more lines of systemic therapy.	Not Approved
69	Adagrasib	2024/6/21	With cetuximab, indicated for the treatment of adult patients with KRAS G12C-mutated locally advanced or metastatic colorectal cancer (CRC), as determined by an FDA-approved test, who have received prior treatment with fluoropyrimidine-, oxaliplatin-, and irinotecan-based chemotherapy.	Not Approved
70	Repotrectinib	2024/6/13	For the treatment of adult and pediatric patients 12 years of age and older with solid tumors that have a neurotrophic tyrosine receptor kinase (NTRK) gene fusion, are locally advanced or metastatic, or where surgical resection is likely to result in severe morbidity, and that have progressed following treatment or have no satisfactory alternative therapy.	Not Approved
71	Tarlatamab-dlle	2024/5/16	For the treatment of patients with extensive stage small cell lung cancer (ES-SCLC) with disease progression on or after platinum-based chemotherapy.	Not Approved
72	Lisocabtagene maraleucel	2024/5/15	For the treatment of adult patients with relapsed or refractory follicular lymphoma (FL) who have received two or more prior lines of systemic therapy.	Not Approved
73	Tovorafenib	2024/4/23	For the treatment of patients 6 months of age and older with relapsed or refractory pediatric low-grade glioma (LGG) harboring a BRAF	Not Approved

			fusion or rearrangement, or BRAF V600 mutation.	
74	Fam-trastuzumab deruxtecan-nxki	2024/4/5	For the treatment of adult patients with unresectable or metastatic HER2-positive (IHC 3+) solid tumors who have received prior systemic therapy and have no satisfactory alternative treatment options.	Not Approved
75	Lifileucel	2024/2/16	For the treatment of adult patients with unresectable or metastatic melanoma previously treated with a PD-1 blocking antibody, and if BRAF V600 mutation positive, a BRAF inhibitor with or without a MEK inhibitor.	Not Approved
76	Pirtobrutinib	2023/12/1	For the treatment of adult patients with chronic lymphocytic leukemia (CLL) or small lymphocytic lymphoma (SLL) who have received at least two prior lines of therapy, including a BTK inhibitor and a BCL-2 inhibitor.	Not Approved
77	Epcoritamab-bysp	2023/5/19	For the treatment of adult patients with relapsed or refractory diffuse large B-cell lymphoma (DLBCL), not otherwise specified, including DLBCL arising from indolent lymphoma, and high-grade B-cell lymphoma after two or more lines of systemic therapy.	Not Approved
78	Retifanlimab-dlwr	2023/3/22	For the treatment of adult patients with metastatic or recurrent locally advanced Merkel cell carcinoma (MCC).	Not Approved
79	Tucatinib	2023/1/19	In combination with trastuzumab is indicated for the treatment of adult patients with RAS wild-type, HER2-positive, unresectable or metastatic colorectal cancer that has progressed following treatment with fluoropyrimidine-, oxaliplatin-, and irinotecan-based chemotherapy.	Not Approved
80	Adagrasib	2022/12/12	For the treatment of adult patients with KRAS G12C-mutated locally advanced or metastatic non-small cell lung cancer (NSCLC), as determined by an FDA-approved test, who have received at least one prior systemic therapy.	Not Approved
81	Tfutibatinib	2022/9/30	For the treatment of adult patients with previously treated, unresectable locally advanced or metastatic intrahepatic cholangiocarcinoma (CCA) harboring fibroblast growth factor receptor 2 (FGFR2) gene fusions or other rearrangements.	Not Approved

82	Selpercatinib	2022/9/21	For the treatment of adult patients with locally advanced or metastatic solid tumors with a RET gene fusion that have progressed on or after prior systemic therapy or who have no satisfactory alternative treatment options.	Not Approved
83	Dabrafenib	2022/6/22	In combination with trametinib, for the treatment of adult and pediatric patients aged 6 years and older with unresectable or metastatic solid tumors with BRAF V600E mutation who have progressed following prior therapy and have no satisfactory alternative treatment options.	Not Approved
84	Trametinib	2022/6/22	In combination with dabrafenib, for the treatment of adult and pediatric patients aged 6 years and older with unresectable or metastatic solid tumors with BRAF V600E mutation who have progressed following prior therapy and have no satisfactory alternative treatment options. The initial AA indication was modified to include the expanded population.	Not Approved
85	Tisagenlecleu cel	2022/5/27	For the treatment of adult patients with relapsed or refractory (r/r) follicular lymphoma (FL) after two or more lines of systemic therapy.	Not Approved
86	Alpelisib	2022/4/5	For the treatment of adult and pediatric patients 2 years of age and older with severe PIK3CA-related overgrowth spectrum (PROS) who require systemic therapy.	Not Approved
87	Zanubrutinib	2021/9/14	For the treatment of adult patients with relapsed or refractory marginal zone lymphoma (MZL) who have received at least one anti-CD20-based regimen.	Not Approved
88	Dostarlimab-gxly	2021/8/17	For the treatment of adult patients with mismatch repair deficient (dMMR) recurrent or advanced solid tumors, as determined by an FDA-approved test, that have progressed on or following prior therapy and who have no satisfactory alternative treatment options.	Not Approved
89	Sotorasib	2021/5/28	For the treatment of adult patients with KRAS G12C-mutated locally advanced or metastatic non-small cell lung cancer (NSCLC), as determined by an FDA-approved test, who have received at least one prior systemic therapy.	Not Approved

90	Axicabtagene ciloleucel	2021/3/5	For the treatment of adult patients with relapsed or refractory follicular lymphoma (FL) after two or more lines of systemic therapy.	Not Approved
91	Brexucabtagene autoleucel	2020/7/24	For the treatment of adult patients with relapsed or refractory mantle cell lymphoma (MCL).	Not Approved
92	Pembrolizumab	2020/6/16	For the treatment of adult and pediatric patients with unresectable or metastatic tumor mutational burden-high (TMB-H) [≥10 mutations/megabase (mut/Mb)] solid tumors, as determined by an FDA-approved test, that have progressed following prior therapy and who have no satisfactory alternative treatment options.	Not Approved
93	Rucaparib	2020/5/15	For the treatment of adult patients with metastatic castration-resistant prostate cancer (mCRPC) associated with a deleterious BRCA mutation (germline and/or somatic) who have been treated with androgen receptor-directed therapy and a taxane-based chemotherapy.	Not Approved
94	Pomalidomide	2020/5/14	For the treatment of patients with AIDS-related Kaposi sarcoma (KS) after failure of highly active antiretroviral therapy (HAART).	Not Approved
95	Pomalidomide	2020/5/14	For the treatment of patients with Kaposi sarcoma (KS) in HIV-negative patients.	Not Approved
96	Ipilimumab	2020/3/10	In combination with nivolumab, for the treatment of patients with hepatocellular carcinoma (HCC) who have been previously treated with sorafenib.	Not Approved
97	Nivolumab	2020/3/10	In combination with ipilimumab, for the treatment of patients with hepatocellular carcinoma (HCC) who have been previously treated with sorafenib.	Regular Approval
98	Tazemetostat	2020/1/23	For the treatment of adult and pediatric patients aged 16 years and older with metastatic or locally advanced epithelioid sarcoma not eligible for complete resection.	Not Approved
99	Nivolumab	2018/7/10	In combination with ipilimumab, for the treatment of adult and pediatric patients 12 years and older with microsatellite instability-high (MSI-H) or mismatch repair deficient (dMMR) metastatic colorectal cancer (CRC) that has progressed following treatment with fluoropyrimidine, oxaliplatin, and irinotecan.	Regular Approval

100	Ipilimumab	2018/7/10	In combination with nivolumab, for the treatment of adult and pediatric patients 12 years and older with microsatellite instability-high (MSI-H) or mismatch repair deficient (dMMR) metastatic colorectal cancer (CRC) that has progressed following treatment with fluoropyrimidine, oxaliplatin, and irinotecan.	Regular Approval
101	Nivolumab	2017/7/31	For the treatment of adult and pediatric patients 12 years and older with microsatellite instability-high (MSI-H) or mismatch repair deficient (dMMR) metastatic colorectal cancer (CRC) that has progressed following treatment with fluoropyrimidine, oxaliplatin, and irinotecan.	Regular Approval
102	Nivolumab	2017/4/25	For the treatment of adult patients with classical Hodgkin lymphoma (cHL) that has relapsed or progressed following: autologous hematopoietic stem cell transplant (HSCT) and brentuximab vedotin, or 3 or more lines of systemic therapy including autologous HSCT.	Not Approved
103	Nivolumab	2016/5/17	For the treatment of patients with classical Hodgkin lymphoma (cHL) that has relapsed or progressed following autologous hematopoietic stem cell transplant (HSCT) and post-transplant brentuximab vedotin.	Not Approved
104	Belinostat	2014/7/3	For the treatment of patients with relapsed or refractory peripheral T-cell lymphoma (PTCL).	Not Approved
105	Infigratinib	2021/5/28	For the treatment of adults with previously treated, unresectable locally advanced or metastatic cholangiocarcinoma with a fibroblast growth factor receptor 2 (FGFR2) fusion or other rearrangement, as detected by an FDA-approved test.	Not Approved
106	Sacituzumab govitecan-hziy	2021/4/13	For the treatment of adult patients with locally advanced or metastatic urothelial cancer (mUC) who have previously received platinum-containing chemotherapy and a programmed death receptor-1 (PD-1) or programmed death ligand 1 (PD-L1) inhibitor.	Not Approved
107	Melphalan flufenamide	2021/2/26	Melphalan flufenamide (Pepaxto, Oncopeptides AB) in combination with dexamethasone is indicated for the treatment of adult patients with relapsed or refractory multiple myeloma who have received at least four prior lines of therapy and whose disease is refractory to at least one proteasome	Not Approved

			inhibitor, one immunomodulatory agent, and one CD38-directed monoclonal antibody.	
108	Uumbralisib	2021/2/5	For the treatment of adult patients with relapsed or refractory marginal zone lymphoma (MZL) who have received at least one prior anti-CD20-based regimen.	Not Approved
109	Umbralisib	2021/2/5	For the treatment of adult patients with relapsed or refractory follicular lymphoma (FL) who have received at least three prior lines of systemic therapy.	Not Approved
110	Pembrolizuma b	2019/6/17	For the treatment of patients with metastatic small cell lung cancer (SCLC) with disease progression on or after platinum-based chemotherapy and at least one other prior line of therapy.	Not Approved
111	Nivolumab	2018/8/16	For the treatment of patients with metastatic small cell lung cancer (SCLC) with disease progression after platinum-based chemotherapy and at least one other prior line of therapy.	Not Approved
112	Pembrolizuma b	2017/9/22	For the treatment of patients with recurrent or locally advanced or metastatic gastric or gastroesophageal junction (GEJ) adenocarcinoma whose tumors express PD-L1 [CPS ≥ 1], as determined by an FDA-approved test, with disease progression on or after two or more prior lines of therapy including fluoropyrimidine- and platinum-containing chemotherapy and if appropriate, HER2/neu-targeted therapy.	Not Approved
113	Nivolumab	2017/9/22	For the treatment of patients with hepatocellular carcinoma (HCC) previously treated with sorafenib.	Regular Approval
114	Durvalumab	2017/5/1	For the treatment of patients with locally advanced or metastatic urothelial carcinoma who have disease progression during or following platinum-containing chemotherapy, or within 12 months of neoadjuvant or adjuvant treatment with platinum-containing chemotherapy.	Not Approved
115	Atezolizumab	2017/4/17	For the treatment of patients with locally advanced or metastatic urothelial carcinoma (mUC) whose disease has progressed during or following platinum-containing chemotherapy, and for the treatment of patients with locally advanced or metastatic urothelial carcinoma (mUC) whose disease has worsened within 12 months of receiving	Not Approved

			platinum-containing chemotherapy before or after surgery.	
116	Ibrutinib	2017/1/18	For the treatment of adult patients with marginal zone lymphoma (MZL) who require systemic therapy and have received at least one prior anti-CD20-based therapy.	Not Approved
117	Atezolizumab	2016/5/18	For the treatment of patients with locally advanced or metastatic urothelial carcinoma who have disease progression during or following platinum-containing chemotherapy, or within 12 months of neoadjuvant or adjuvant treatment with platinum-containing chemotherapy.	Not Approved
118	Idelalisib	2014/7/23	For the treatment of patients with relapsed follicular B-cell non-Hodgkin lymphoma (FL) who have received at least two prior systemic therapies, and for the treatment of patients with relapsed small lymphocytic lymphoma (SLL) who have received at least two prior systemic therapies.	Not Approved
119	Ibrutinib	2013/11/13	For the treatment of adult patients with mantle cell lymphoma (MCL) who have received at least one prior therapy.	Regular Approval
120	Vincristine sulfate liposomal	2012/8/9	For the treatment of adults with Philadelphia chromosome-negative (Ph-) acute lymphoblastic leukemia (ALL) in second or greater relapse, or whose disease has progressed following two or more anti-leukemia lines of therapy.	Not Approved
121	Romidepsin	2011/6/16	For the treatment of patients with peripheral T-cell lymphoma (PTCL) who have received at least one prior therapy.	Not Approved
122	Tositumomab and iodine i 131 tositumomab	2004/12/22	For the treatment of patients with relapsed or refractory low-grade, follicular, or transformed CD20-positive non-Hodgkin lymphoma (NHL) who have not received rituximab.	Not Approved
123	Gefitinib	2003/5/5	As monotherapy for the treatment of patients with locally advanced or metastatic non-small cell lung cancer (NSCLC) after failure of both platinum-based and docetaxel chemotherapies.	Regular Approval

Supplementary Table 3. Status of drugs conditionally approved only in China

Number	Drugs with Conditional Approval Only in the China	Approval Date	Indication	Approval Status in US
1	Tislelizumab	2021/6/22	For the treatment of hepatocellular carcinoma (HCC) that has been previously treated with at least one systemic therapy.	Not Approved
2	Tislelizumab	2020/4/9	For the treatment of locally advanced or metastatic urothelial carcinoma with high PD-L1 expression that has progressed on or following platinum-containing chemotherapy, including within 12 months of neoadjuvant or adjuvant chemotherapy.	Not Approved
3	Tislelizumab	2022/3/8	This product is indicated for adult patients with advanced solid tumors that are unresectable or metastatic and are microsatellite instability-high (MSI-H) or mismatch repair deficient (dMMR): patients with advanced colorectal cancer that has progressed following prior treatment with fluoropyrimidine, oxaliplatin, and irinotecan; and patients with other advanced solid tumors that have progressed following prior therapy and have no satisfactory alternative treatment options.	Not Approved
4	Socazolimab	2023/12/19	For the treatment of recurrent or metastatic cervical cancer.	Not Approved
5	Camrelizumab	2020/3/3	For the treatment of patients with relapsed or refractory classical Hodgkin lymphoma who have received at least two prior lines of systemic chemotherapy.	Not Approved
6	Camrelizumab	2021/4/27	For the treatment of patients with advanced nasopharyngeal carcinoma who have disease progression or intolerance after receiving two or more prior lines of chemotherapy.	Not Approved
7	Pucotenlimab	2022/9/20	This product is indicated for patients with unresectable or metastatic melanoma who have failed prior systemic therapy.	Not Approved
8	Pucotenlimab	2022/7/19	This product is indicated for adult patients with advanced solid tumors that are unresectable or metastatic and are microsatellite instability-high (MSI-H) or mismatch repair deficient (dMMR): patients with advanced colorectal cancer that has progressed following prior treatment with fluoropyrimidine, oxaliplatin, and irinotecan;	Not Approved

			and patients with other advanced solid tumors that have progressed following at least one prior line of therapy and have no satisfactory alternative treatment options.	
9	Toripalimab	2021/2/10	This product is indicated for the treatment of recurrent/metastatic nasopharyngeal carcinoma that has failed after two or more prior lines of systemic therapy.	Regular Approval
10	Toripalimab	2021/4/7	Toripalimab is indicated for the treatment of locally advanced or metastatic urothelial carcinoma that has progressed on or following platinum-containing chemotherapy, including within 12 months of neoadjuvant or adjuvant chemotherapy.	Not Approved
11	Disitamab	2021/6/8	This product is indicated for patients with HER2-overexpressing locally advanced or metastatic gastric cancer (including gastroesophageal junction adenocarcinoma) who have received at least two prior systemic chemotherapy regimens. HER2 overexpression is defined as an immunohistochemistry (IHC) result of 2+ or 3+.	Not Approved
12	Disitamab	2021/12/31	This product is indicated for patients with locally advanced or metastatic urothelial carcinoma who have received prior platinum-containing chemotherapy and have HER2 overexpression. HER2 overexpression is defined as an immunohistochemistry (IHC) result of 2+ or 3+.	Not Approved
13	Zimberelimab	2023/6/30	This product is indicated for the treatment of recurrent or metastatic cervical cancer with PD-L1 expression positive (CPS≥1) that has progressed after one or more prior lines of standard platinum-containing chemotherapy.	Not Approved
14	Zimberelimab	2021/8/25	This product is indicated for adult patients with relapsed or refractory classical Hodgkin lymphoma who have received at least two prior lines of systemic chemotherapy.	Not Approved
15	Ivosidenib	2022/1/30	This product is indicated for adult patients with relapsed or refractory acute myeloid leukemia (AML) diagnosed with a susceptible isocitrate dehydrogenase-1 (IDH1) mutation as detected by a fully validated test.	Regular Approval
16	Serplulimab	2022/3/22	This product is indicated for adult patients with advanced solid tumors that are unresectable or metastatic and are microsatellite instability-high (MSI-H):	Not Approved

			patients with advanced colorectal cancer that has progressed following prior treatment with fluoropyrimidine, oxaliplatin, and irinotecan; patients with advanced gastric cancer that has progressed following at least two prior lines of therapy and have no satisfactory alternative treatment options; and patients with other advanced solid tumors that have progressed following at least one prior line of therapy and have no satisfactory alternative treatment options.	
17	Cadonilimab	2022/6/28	This product is indicated for the treatment of patients with recurrent or metastatic cervical cancer that has failed prior platinum-containing chemotherapy.	Not Approved
18	Envafolimab	2021/11/24	This product is indicated for adult patients with advanced solid tumors that are unresectable or metastatic and are microsatellite instability-high (MSI-H) or mismatch repair deficient (dMMR): patients with advanced colorectal cancer that has progressed following prior treatment with fluoropyrimidine, oxaliplatin, and irinotecan; and patients with other advanced solid tumors that have progressed following prior therapy and have no satisfactory alternative treatment options.	Not Approved
19	Mitoxantrone	2022/1/7	This product is indicated for patients with relapsed or refractory peripheral T-cell lymphoma (PTCL) who have received at least one prior line of standard therapy.	Not Approved
20	Relmacabtagene	2021/9/1	This product is indicated for the treatment of adult patients with relapsed or refractory large B-cell lymphoma after two or more lines of systemic therapy, including diffuse large B-cell lymphoma not otherwise specified, diffuse large B-cell lymphoma transformed from follicular lymphoma, grade 3b follicular lymphoma, primary mediastinal large B-cell lymphoma, and high-grade B-cell lymphoma with MYC and BCL-2 and/or BCL-6 rearrangements (double-hit/triple-hit lymphoma).	Not Approved
21	Relmacabtagene	2022/9/30	This product is indicated for the treatment of adult patients with relapsed or refractory follicular lymphoma (including histological grades 1, 2, and 3a) after two or more lines of systemic therapy.	Not Approved
22	Savolitinib	2021/6/22	This product is indicated for adult patients with locally advanced or metastatic non-small cell lung cancer (NSCLC) harboring mesenchymal-epithelial transition (MET) exon 14 skipping alterations whose disease has progressed on or after platinum-	Not Approved

			containing chemotherapy, or who are intolerant to standard platinum-containing chemotherapy.	
23	Sugemalimab	2023/10/27	This product as a single agent is indicated for the treatment of relapsed or refractory extranodal NK/T-cell lymphoma (R/R ENKTL).	Not Approved
24	Ensartinib	2020/11/17	This product is indicated for patients with anaplastic lymphoma kinase (ALK)-positive locally advanced or metastatic non-small cell lung cancer (NSCLC) whose disease has progressed after prior crizotinib therapy or who are intolerant to crizotinib.	Regular Approval
25	Orelabrutinib	2023/4/17	This product is indicated for adult patients with marginal zone lymphoma (MZL) who have received at least one prior therapy.	Not Approved
26	Orelabrutinib	2020/12/25	This product is indicated for adult patients with chronic lymphocytic leukemia (CLL)/small lymphocytic lymphoma (SLL) who have received at least one prior therapy.	Not Approved
27	Orelabrutinib	2020/12/25	This product is indicated for adult patients with mantle cell lymphoma (MCL) who have received at least one prior therapy.	Not Approved
28	Zanubrutinib	2020/6/2	This product is indicated for adult patients with chronic lymphocytic leukemia (CLL)/small lymphocytic lymphoma (SLL) who have received at least one prior therapy.	Regular Approval
29	Zanubrutinib	2021/6/16	This product is indicated for adult patients with Waldenström's macroglobulinemia (WM) who have received at least one prior therapy.	Regular Approval
30	Zanubrutinib	2024/5/8	This product, in combination with obinutuzumab, is indicated for adult patients with relapsed or refractory follicular lymphoma (FL) who have received at least two prior lines of systemic therapy.	Conditional Approval, based on randomized controlled trial
31	Entrectinib	2024/2/6	This product is indicated for the treatment of pediatric solid tumors.	Not Approved
32	Emapalumab	2022/3/8	This product is indicated for adult and pediatric (neonate and above) patients with primary hemophagocytic lymphohistiocytosis (HLH) that is refractory, recurrent, progressive, or intolerant to conventional therapy.	Regular Approval

33	Glumetinib	2023/3/7	This product is indicated for the treatment of locally advanced or metastatic non-small cell lung cancer (NSCLC) harboring mesenchymal-epithelial transition (MET) exon 14 skipping alterations.	Not Approved
34	Pamiparib	2021/4/30	This product is indicated for the treatment of patients with recurrent advanced ovarian cancer, fallopian tube cancer, or primary peritoneal cancer with germline BRCA (gBRCA) mutations who have received two or more prior lines of chemotherapy.	Not Approved
35	Almonertinib	2020/3/17	This product is indicated for the treatment of adult patients with locally advanced or metastatic non-small cell lung cancer (NSCLC) whose disease has progressed during or after epidermal growth factor receptor (EGFR) tyrosine kinase inhibitor (TKI) therapy and who have a positive EGFR T790M mutation status confirmed by testing.	Not Approved
36	Dabrafenib	2022/3/22	This product, in combination with trametinib, is indicated for the treatment of patients with BRAF V600 mutation-positive metastatic non-small cell lung cancer (NSCLC).	Regular Approval
37	Trametinib	2022/3/22	This product, in combination with dabrafenib mesylate, is indicated for the treatment of patients with BRAF V600 mutation-positive metastatic non-small cell lung cancer (NSCLC).	Regular Approval
38	Fluzoparib	2020/12/11	This product is indicated for patients with platinum-sensitive recurrent ovarian cancer, fallopian tube cancer, or primary peritoneal cancer with germline BRCA mutation (gBRCAm) who have received two or more prior lines of chemotherapy.	Not Approved
39	Furmonertinib	2021/3/2	This product is indicated for the treatment of adult patients with locally advanced or metastatic non-small cell lung cancer (NSCLC) whose disease has progressed during or after epidermal growth factor receptor (EGFR) tyrosine kinase inhibitor (TKI) therapy and who have a confirmed positive EGFR T790M mutation status by testing.	Not Approved
40	Larotrectinib	2022/6/23	Larotrectinib sulfate oral solution is indicated for the treatment of adult and pediatric patients with solid tumors that:are diagnosed with a neurotrophic tyrosine receptor kinase (NTRK) gene fusion without an acquired resistance mutation, as detected by a fully validated test,are locally advanced, metastatic, or where surgical resection is	Not Approved

			likely to result in severe morbidity, andhave no satisfactory alternative treatments or have progressed following prior therapy.	
41	Bozitinib	2023/11/17	This product is indicated for adult patients with locally advanced or metastatic non-small cell lung cancer (NSCLC) harboring mesenchymal-epithelial transition (MET) exon 14 skipping alterations.	Not Approved
42	Olverembatinib	2021/11/24	This product is indicated for the treatment of adult patients with chronic phase or accelerated phase chronic myeloid leukemia (CML) resistant to any tyrosine kinase inhibitor (TKI) and diagnosed with a T315I mutation as detected by a fully validated test.	Not Approved
43	Linperlisib	2022/11/8	This product is indicated for adult patients with relapsed or refractory follicular lymphoma who have received at least two prior systemic therapies.	Not Approved
44	Inaticabtagene	2023/11/7	This product is indicated for the treatment of adult patients with relapsed or refractory B-cell acute lymphoblastic leukemia.	Not Approved
45	Avapritinib	2021/3/30	This product is indicated for the treatment of adult patients with unresectable or metastatic gastrointestinal stromal tumors (GIST) harboring platelet-derived growth factor receptor alpha (PDGFRA) exon 18 mutations, including PDGFRA D842V mutations.	Regular Approval
46	Narlumosbart	2023/9/5	This product is indicated for the treatment of giant cell tumor of bone that is unresectable or where surgery is difficult.	Not Approved
47	Penpulimab	2021/8/3	This product is indicated for adult patients with relapsed or refractory classical Hodgkin lymphoma who have received at least two prior lines of systemic chemotherapy.	Not Approved
48	Equecabtagene	2023/6/30	This product is indicated for adult patients with relapsed or refractory multiple myeloma who have received three or more prior lines of systemic therapy.	Not Approved
49	Repotrectinib	2024/5/8	This product is indicated for adult patients with ROS1-positive locally advanced or metastatic non-small cell lung cancer (NSCLC).	Regular Approval
50	Golidocitinib	2024/6/18	This product as a single agent is indicated for adult patients with relapsed or refractory peripheral T-cell lymphoma (r/r PTCL) who	Not Approved

			have received at least one prior line of systemic therapy.	
51	Enlonstobart	2024/6/25	This product is intended for the treatment of patients with recurrent or metastatic cervical cancer with positive PD-L1 expression who have failed at least one prior line of platinum-containing chemotherapy.	Not Approved
52	Fulzerasib	2024/8/20	This product is indicated for adult patients with advanced non-small cell lung cancer (NSCLC) harboring the Kirsten rat sarcoma viral oncogene homolog G12C (KRAS G12C) mutation who have received at least one prior systemic therapy.	Not Approved
53	Garsorasib	2024/11/5	This product is indicated for the treatment of locally advanced or metastatic non-small cell lung cancer (NSCLC) with confirmed KRAS G12C mutation that has progressed after or is intolerant to prior first-line systemic therapy.	Not Approved
54	Tunlametinib	2024/3/12	This product is intended for the treatment of adult patients with advanced melanoma harboring NRAS mutations who have previously received immunotherapy.	Not Approved
55	iparomlimab and tuvonralimab	2024/9/26	This product is indicated for the treatment of patients with recurrent or metastatic cervical cancer who have failed prior platinum-containing chemotherapy.	Not Approved
56	Ciltacabtagene Autoleucel	2024/8/20	This product is indicated for the treatment of adult patients with relapsed or refractory multiple myeloma after prior therapy with a proteasome inhibitor and an immunomodulatory agent.	Regular Approval
57	Trastuzumab	2024/8/5	This product is indicated for adult patients with HER2-positive gastric or gastroesophageal junction adenocarcinoma.	Regular Approval
58	Trastuzumab	2024/10/9	This product is indicated for adult patients with unresectable locally advanced or metastatic non-small cell lung cancer (NSCLC) harboring HER2 (ERBB2) activating mutations who have received at least one prior systemic therapy.	Regular Approval
59	Zevorcabtagene Autoleucel	2024/2/23	This product is indicated for the treatment of adult patients with relapsed or refractory multiple myeloma who have progressed after at least three prior lines of therapy.	Not Approved

60	Acalabrutinib Maleate	2024/10/29	This product is indicated for adult patients with mantle cell lymphoma (MCL) who have received at least one prior therapy.	Regular Approval
61	taletrectinib	2024/12/17	This product is indicated for adult patients with ROS1-positive locally advanced or metastatic non-small cell lung cancer (NSCLC) who have progressed after prior ROS1-TKI therapy.	Regular Approval
62	Fruguintinib	2024/12/1	This product, in combination with Sintilimab injection, is indicated for patients with advanced mismatch repair proficient (pMMR) endometrial cancer who have failed prior systemic anti-tumor therapy and are not candidates for curative surgery or curative radiotherapy.	Not Approved
63	Dinutuximab	2021/8/12	This product is indicated for the treatment of neuroblastoma.	Regular Approval
64	Lisaftoclax	2025/7/8	This product is indicated for adult patients with chronic lymphocytic leukemia (CLL)/small lymphocytic lymphoma (SLL) who have received at least one prior systemic therapy including a Bruton's tyrosine kinase (BTK) inhibitor.	Not Approved
65	SHR2554	2025/8/26	This product is indicated for adult patients with relapsed or refractory peripheral T-cell lymphoma who have received at least one prior line of systemic therapy.	Not Approved
66	Bozitinib	2025/6/24	This product is indicated for the treatment of patients with locally advanced or metastatic non-small cell lung cancer (NSCLC) with mesenchymal-epithelial transition (MET) amplification.	Not Approved
67	Glecirasib	2025/5/20	This product is indicated for adult patients with advanced non-small cell lung cancer (NSCLC) harboring the Kirsten rat sarcoma viral oncogene homolog (KRAS) G12C mutation who have received at least one prior systemic therapy.	Not Approved
68	trastuzumab rezetecan	2025/5/27	This product as a single agent is indicated for the treatment of adult patients with unresectable locally advanced or metastatic non-small cell lung cancer (NSCLC) harboring HER2 (ERBB2) activating mutations who have received at least one prior systemic therapy.	Not Approved

69	Ifupinostat	2025/6/30	This product as a single agent is indicated for adult patients with relapsed or refractory diffuse large B-cell lymphoma who have received at least two prior lines of systemic therapy.	Not Approved

Notes:
The 36 anticancer drugs in China and the 38 in the United States that received conditional or accelerated approvals based on single-arm trials were all the same for the same indication, Among them, Pralsetinib and Selpercatinib were conditionally approved for the treatment of thyroid cancer and medullary thyroid carcinoma in China, and both indications were approved as one approval, whereas the two indications received accelerated approval in the United States, which were two approvals, the rest of the approvals were one-to-one correspondence. Therefore, in addition to the 36 drugs concurrently marketed in both China and the United States, there are 69 oncology drugs approved only in China.

Supplementary Table 4. Approval requirements for conditional approval procedures in China and the United States

Requirements	China (1,2,3)	United States (3,4)
Timing for Application	During the drug clinical trial period	Discussing the possibility of accelerated approval during clinical development
Applicable Objects	First marketing or new indications	First marketing or new indications
Technical Requirements	1. Drugs for treating serious life-threatening diseases with no effective treatment, where clinical trial data have demonstrated efficacy and can predict clinical value;<br>2. Drugs urgently needed for public health, where clinical trial data have shown efficacy and can predict clinical value;<br>3. Vaccines urgently needed for responding to major public health emergencies or other vaccines deemed urgently needed by the National Health Commission, where the benefits are assessed to outweigh the risks.	1. Treating serious diseases;<br>2. Having clear advantages over existing therapies;<br>3. Demonstrating a therapeutic effect on a surrogate endpoint that reasonably predicts clinical benefit, or on an intermediate clinical endpoint that can be measured before irreversible morbidity or mortality (IMM) and reasonably predicts the drug's effect on IMM or other clinical benefits.
Approval Evidence	Surrogate endpoints, intermediate clinical endpoints, or early clinical trial data	Surrogate endpoints or intermediate clinical endpoints
Post-Marketing Requirements	Confirmatory clinical studies must be completed after marketing to demonstrate that the benefits outweigh the risks	Confirmatory clinical studies must be completed after marketing to demonstrate that the benefits outweigh the risks
Withdrawal Mechanism	If post-marketing studies fail to demonstrate that the benefits outweigh the risks, or if the applicant fails to complete the required studies and submit a supplementary application within the	1. Post-marketing studies fail to confirm clinical benefit;<br>2. The applicant fails to diligently fulfill post-marketing study obligations;<br>3. Restrictions are insufficient to ensure

	specified timeframe, the CDE will revoke the drug registration certificate in accordance with the procedures.	the safe use of the drug;<br>4. The applicant fails to comply with agreed-upon post-marketing restrictions;<br>5. Promotional materials are false or misleading;<br>6. Other evidence indicates that the drug has not been shown to be safe or effective under its conditions of use.
Validity Period	The validity period of the conditional approval drug registration certificate is specified in the certificate	Not specified

Supplementary Table 5. Results of meta-analysis for subgroups of indications in the United States and China

Subgroup	RR (95% CI) in the US	I^2（%）	RR (95% CI) in China	I^2（%）
Classification of Indications	P＜0.001		P＜0.001	
Lymphoma	57.6(52.3-63.6)	94.1	61.5(54.9-68.8)	90.6
Lung Cancer	40.4(35.7-45.8)	87.9	60.7(55.7-66.0)	79.3
Leukemia	43.4(36.3-51.8)	93.7	61.0(51.1-72.8)	82.2
Myeloma	39.8(29.3-54.1)	95.0	64.1(53.3-77.1）	95.6
Solid Tumor	47.6(39.8-56.9)	84.7	56.5(48.2-66.2)	81.1
Urothelial Cancer	28.9(20.3-41.2)	96.6	32.5(21.1-50.1)	82.3
Colorectal Cancer	29.6(21.2-41.3)	87.5	-1	-1
Ovarian Cancer	33.4(23.0-48.6)	89.1	55.1(39.0-77.9)	91.3
Hepatocellular carcinoma	22.5(14.5-34.9)	75.7	-2	-2
Other	36.5(31.8-41.9)	87.5	36.4(28.2-47.0)	95.1
Pooled effect value	40.6(38.0-43.5)	94.7	52.6(49.2-56.2)	93.1

Notes:
Two approvals in the United States and one approval in China had RR values that could not be retrieved and were therefore excluded from the analysis
1 The pooled effect value for myeloma in China intersects the null line and is not statistically significant
2 The amount of data is too low.

Supplementary Table 6. RR values of single-arm trials for the same drug in China and the United States for the same indication

sequence number	Product name	Indications	RR (95% CI) in China	RR (95% CI) in the US
1	Selinexor	Myeloma	0.29(0.20,0.40)	0.25(0.16,0.36)
2	Pralatrexate	Lymphoma	0.52(0.40,0.64)	0.27(0.19,0.36)

3	Mobocertinib	Non-Small Cell Lung Cancer	0.28(0.2,0.37)	0.28(0.20,0.37)
4	Blinatumomab	Leukemia	0.33(0.22,0.45)	0.85(0.74,0.93)
5	Blinatumomab	Leukemia	0.48(0.35,0.6)	0.32(0.26,0.40)
6	Sacituzumab	Breast Cancer	0.39(0.28,0.50)	0.33(0.25,0.43)
7	Carfilzomib	Myeloma	0.36(0.27,0.45)	0.23(0.18,0.29)
8	Pemigatinib	Cholangiocarcinoma	0.50(0.31,0.69)	0.36(0.27,0.45)
9	Naxitamab	Neuroblastoma	0.34(0.20,0.51)	0.34(0.20,0.51)
10	Duvelisib	Lymphoma	0.86(0.65,0.97)	0.42(0.31,0.54)
11	Copanlisib	Lymphoma	0.50(0.21,0.79)	0.59(0.49,0.68)
12	Pralsetinib	Thyroid Cancer	0.73(0.52,0.88)	0.60(0.46,0.73)
13	Pralsetinib	Medullary Thyroid Cancer	0.73(0.52,0.88)	0.60(0.46,0.73)
14	Pralsetinib	Non-Small Cell Lung Cancer	0.56(0.38,0.74)	0.57(0.46,0.68)
15	Glofitamab	Lymphoma	0.52(0.32,0.71)	0.56(0.47,0.65)
16	Zanubrutinib	Lymphoma	0.84 (0.74,0.91)	0.84 (0.74,0.91)
17	Entrectinib	Solid Tumor	0.64(0.52,0.75)	0.70(0.51,0.84)
18	Selpercatinib	Non-Small Cell Lung Cancer	0.69(0.48,0.86)	0.64(0.54,0.73)
19	Selpercatinib	Thyroid Cancer	0.58(0.37,0.77)	0.79(0.54,0.94)
20	Selpercatinib	Medullary Thyroid Cancer	0.58(0.37,0.77)	0.73(0.62,0.82)
21	Lorlatinib	Non-Small Cell Lung Cancer	0.70(0.58,0.81)	0.48(0.42,0.55)
22	Acalabrutinib	Lymphoma	0.82(0.66,0.93)	0.81(0.73,0.87)
23	Larotrectinib	Solid Tumor	0.72(0.65,0.79)	0.75(0.61,0.85)
24	Selinexor	Lymphoma	0.21(0.12,0.34)	0.29(0.22,0.38)
25	Teclistamab	Myeloma	0.63(0.55,0.70)	0.62(0.52,0.71)
26	Pirtobrutinib	Lymphoma	0.63(0.45,0.79)	0.50(0.41,0.59)
27	Pembrolizumab	Solid Tumor	0.70(0.46,0.88)	0.40(0.32,0.48)
28	Mirvetuximab Soravtansine	Ovarian Cancer	0.32(0.23,0.42)	0.32(0.23,0.42)
29	Mosunetuzumab	Lymphoma	0.78(0.75,0.81)	0.80(0.70,0.88)
30	Loncastuximab Tesirine	Lymphoma	0.52(0.40,0.64)	0.48(0.40,0.57)
31	Lurbinectedin	small cell lung cancer	0.46(0.27,0.65)	0.35(0.26,0.45)
32	Zongertinib	Non-Small Cell Lung Cancer	0.71(0.60,0.80)	0.75(0.63,0.83)
33	Elranatamab	Myeloma	0.61(0.52,0.70)	0.58(0.47,0.68)
34	Talquetamab	Myeloma	0.74(NA,NA)	0.73(0.63,0.81)
35	Zanidatamab	Myeloma	0.42(0.28,0.57)	0.52(0.39,0.65)

36	Sunvozertinib	Non-Small Cell Lung Cancer	0.61(0.50,0.71)	0.46(0.35,0.57)
37	Tazemetostat	Lymphoma	0.64(0.41,0.83)	0.34(0.22,0.48)
38	Tafasitamab	Lymphoma	0.73(NA,NA)	0.55(0.43,0.67)

Supplementary Table 7. Specific situations where confirmatory trials and accelerated pre-approval trials are the same trials in the United States and China

Product name	Accelerated approval time	Regular approval time	Status of confirmatory trials
Nelarabine	10/28/2005	7/31/2019	Supplemental complete trial data
Imatinib mesylate	9/27/2006	4/1/2011	Supplemental complete trial data
Nilotinib	10/29/2007	1/14/2011	24-month follow-up; Increased number of subjects; RR improved from 40% to 51%
Omacetaxine mepesuccinate	10/26/2012	2/10/2014	24-month follow-up
Ponatinib	12/14/2012	11/28/2016	An RR end point measurement was added
Capmatinib	5/6/2020	8/10/2022	Increased number of subjects; RR improved from 41% to 68%
Selpercatinib	5/8/2020	09/21/2022	Increased number of subjects
Pralsetinib	9/4/2020	8/9/2023	Increased number of subjects; RR improved from 57% to 63%
Cemiplimab-rwlc	2/9/2021	4/28/2023	Increased number of subjects; RR improved from 29% to 32%
Dostarlimab-gxly	4/22/2021	2/9/2023	Increased number of subjects; RR improved from 42% to 45%
Selpercatinib	5/8/2020	6/12/2024	Increased number of subjects; RR improved from 79% to 85%
Blinatumomab*	12/2/2020	4/13/2022	Increased number of subjects
Avapritinib*	3/30/2021	2/3/2023	Increased number of subjects; RR improved from 63% to 79%
Pralsetinib*	3/23/2021	6/27/2023	Increased number of subjects; RR improved from 56% to 78%
Savolitinib*	6/22/2021	1/8/2025	Supplemental complete trial data
Dinutuximab*	8/12/2021	8/31/2025	Supplemental complete trial data
Pembrolizumab*	9/5/2023	7/5/2024	Supplemental complete trial data

Note: *Marketed drugs in China

Supplementary Table 8. The confirmatory trial is a reconducted single-arm trial in the United States

Product name	Accelerated approval time	Regular approval time	Status of confirmatory trials
Clofarabine	12/28/2004	7/18/2022	Increased number of subjects
Avelumab	3/23/2017	9/6/2023	Increased number of subjects; RR improved from 33% to 46%
Pembrolizumab	12/19/2018	10/12/2023	Increased number of subjects
Tepotinib	2/3/2021	2/15/2024	Increased number of subjects; RR improved from 43% to 57%

Note: [1]Marketed drugs in China

Supplementary Table 9. Confirmatory trial design and clinical endpoints of anticancer drugs that have been converted to regular approval in China and the United States

Confirmatory trial design	Clinical endpoints	the United States（n=69）No. (%)	China（n=25）No. (%)
Randomized controlled trial	OS	12（17.4）	2（8.0）
	PFS	24（34.8）	13（52.0）
	OS and PFS	12（17.4）	0（0）
	DFS	2（2.9）	0（0）
	TTP	1（1.4）	0（0）
	RR	3（4.3）	2（8.0）
	EFS	0（0）	2（8.0）
Single-arm trial	RR	4（5.8）	0（0）
Same trial	RR	11（16.0）	6（24.0）

Note: OS: Overall Survival; PFS: Progression-free Survival; DFS: Disease-Free Survival; TTP: Time To Progression

2 References

1. NMPA. Provisions for Drug Registration. 2020. Available online at: https://www.cde.org.cn/main/policy/view/2f5d01af26ead0e3b149d99ad8022d8d (Accessed March 8, 2026).
2. Liu H, Balghyn T, Hu CX, et al. Comparative perspective on the optimization path of China's conditional approval policy for drugs. Zhongguo Xinyao Yu Linchuang Zazhi. Published online January 8, 2026. doi:10.3969/j.issn.1007-7669.2026.01.001
3. Qi YL, Zou LM, Jiang YL, et al. Conditional approval policy in China and review standards for innovative anticancer drugs. Zhongguo Xinyao Zazhi. 2023;32(2):189-197.
4. FDA. Guidance for Industry Expedited Programs for Serious Conditions – Drugs and Biologics. 2014. Available online at: https://www.fda.gov/media/119748/download (Accessed March 9, 2026).
